# Supplementary material for: Association of exercise and ADHD symptoms: Analysis within an adult general population sample
Source: PLoS One. 2025 Feb 11;20(2):e0314508. doi: 10.1371/journal.pone.0314508 (PMC11813077; doi:10.1371/journal.pone.0314508)
Supplement: S1 Checklist — (DOCX) [file pone.0314508.s001.docx]

STROBE Statement—checklist of items that should be included in reports of observational studies

|  | Item No. | Recommendation | Page  No. | Relevant text from manuscript |
| --- | --- | --- | --- | --- |
| **Title and abstract** | 1 | (*a*) Indicate the study’s design with a commonly used term in the title or the abstract | 2 | Covered in Abstract: “This cross-sectional study-” |
|  |  | (*b*) Provide in the abstract an informative and balanced summary of what was done and what was found | 2 | Covered in Abstract |
| Introduction | | | |  |
| Background/rationale | 2 | Explain the scientific background and rationale for the investigation being reported | 3-6 | Covered in Introduction |
| Objectives | 3 | State specific objectives, including any prespecified hypotheses | 6-7 | Covered in Introduction |
| Methods | | | |  |
| Study design | 4 | Present key elements of study design early in the paper | 7 | Covered in Design subsection of Method |
| Setting | 5 | Describe the setting, locations, and relevant dates, including periods of recruitment, exposure, follow-up, and data collection | 7-9 | Covered in participants subsection of method |
| Participants | 6 | (*a*) *Cohort study*—Give the eligibility criteria, and the sources and methods of selection of participants. Describe methods of follow-up  *Case-control study*—Give the eligibility criteria, and the sources and methods of case ascertainment and control selection. Give the rationale for the choice of cases and controls  *Cross-sectional study*—Give the eligibility criteria, and the sources and methods of selection of participants | 7-8,11 | Covered in Participants and Procedure subsections of Method |
|  |  | (*b*) *Cohort study*—For matched studies, give matching criteria and number of exposed and unexposed  *Case-control study*—For matched studies, give matching criteria and the number of controls per case |  |  |
| Variables | 7 | Clearly define all outcomes, exposures, predictors, potential confounders, and effect modifiers. Give diagnostic criteria, if applicable | 9-13 | Covered in Materials and Data analysis subsections of Method |
| Data sources/ measurement | 8* | For each variable of interest, give sources of data and details of methods of assessment (measurement). Describe comparability of assessment methods if there is more than one group | *9-12, 28-29* | Covered in Materials subsection of Method and limitations of Discussion |
| Bias | 9 | Describe any efforts to address potential sources of bias | N/A |  |
| Study size | 10 | Explain how the study size was arrived at | 8 | “G-Power calculations indicated that for 95% power, with a rejection criterion of p < .05, and medium effect size (f’ = .15), that 184 participants would be needed for a multiple regression with 12 potential predictors” |

Continued on next page

| Quantitative variables | 11 | Explain how quantitative variables were handled in the analyses. If applicable, describe which groupings were chosen and why |  | 9, 12-13 | Covered in Materials and Data analysis subsections of Method |
| --- | --- | --- | --- | --- | --- |
| Statistical methods | 12 | (*a*) Describe all statistical methods, including those used to control for confounding |  | 12-13 | Covered in Data analysis subsection of Method |
|  |  | (*b*) Describe any methods used to examine subgroups and interactions |  | 12-13 | Covered in Data analysis subsection of Method |
|  |  | (*c*) Explain how missing data were addressed |  | 11 | Covered in Procedure subsection of Method |
|  |  | (*d*) *Cohort study*—If applicable, explain how loss to follow-up was addressed  *Case-control study*—If applicable, explain how matching of cases and controls was addressed  *Cross-sectional study*—If applicable, describe analytical methods taking account of sampling strategy |  | N/A |  |
|  |  | (*e*) Describe any sensitivity analyses |  | N/A |  |
| Results | | | | |  |
| Participants | 13* | (a) Report numbers of individuals at each stage of study—eg numbers potentially eligible, examined for eligibility, confirmed eligible, included in the study, completing follow-up, and analysed |  | 8 | Covered in Participants subsection of Method |
|  |  | (b) Give reasons for non-participation at each stage |  | 8 | Covered in Participants subsection of Method |
|  |  | (c) Consider use of a flow diagram |  | N/A |  |
| Descriptive data | 14* | (a) Give characteristics of study participants (eg demographic, clinical, social) and information on exposures and potential confounders |  | 8,14-15 | Covered in Participants subsection of Method and Descriptive statistics subsection of Results |
|  |  | (b) Indicate number of participants with missing data for each variable of interest |  | N/A | Only participants with completed data for all measures were used in analysis |
|  |  | (c) *Cohort study*—Summarise follow-up time (eg, average and total amount) |  |  |  |
| Outcome data | 15* | *Cohort study*—Report numbers of outcome events or summary measures over time |  |  |  |
|  |  | *Case-control study—*Report numbers in each exposure category, or summary measures of exposure |  |  |  |
|  |  | *Cross-sectional study—*Report numbers of outcome events or summary measures |  | 14-15 | Covered in Descriptive statistics subsection of Results |
| Main results | 16 | (*a*) Give unadjusted estimates and, if applicable, confounder-adjusted estimates and their precision (eg, 95% confidence interval). Make clear which confounders were adjusted for and why they were included |  | 14-21 | SD, IQR, and 95% CIs reported throughout Results |
|  |  | (*b*) Report category boundaries when continuous variables were categorized |  | 9, 12-13 | Covered in Materials and Data analysis subsections of Method |
|  |  | © If relevant, consider translating estimates of relative risk into absolute risk for a meaningful time period |  | N/A |  |

Continued on next page

| Other analyses | 17 | Report other analyses done—eg analyses of subgroups and interactions, and sensitivity analyses |  | 18-21 | Covered throughout Results |
| --- | --- | --- | --- | --- | --- |
| Discussion | | | | |  |
| Key results | 18 | Summarise key results with reference to study objectives |  | 22 | Covered at start of Discussion |
| Limitations | 19 | Discuss limitations of the study, taking into account sources of potential bias or imprecision. Discuss both direction and magnitude of any potential bias |  | 28-30 | Covered in limitations of Discussion |
| Interpretation | 20 | Give a cautious overall interpretation of results considering objectives, limitations, multiplicity of analyses, results from similar studies, and other relevant evidence |  | 22-28 | Covered throughout Discussion |
| Generalisability | 21 | Discuss the generalisability (external validity) of the study results |  | 28-30 | Covered in limitations of Discussion |
| Other information | |  | | |  |
| Funding | 22 | Give the source of funding and the role of the funders for the present study and, if applicable, for the original study on which the present article is based |  | - | No funding was received, specified in submission form |

*Give information separately for cases and controls in case-control studies and, if applicable, for exposed and unexposed groups in cohort and cross-sectional studies.

**Note:** An Explanation and Elaboration article discusses each checklist item and gives methodological background and published examples of transparent reporting. The STROBE checklist is best used in conjunction with this article (freely available on the Web sites of PLoS Medicine at http://www.plosmedicine.org/, Annals of Internal Medicine at http://www.annals.org/, and Epidemiology at http://www.epidem.com/). Information on the STROBE Initiative is available at www.strobe-statement.org.
