# Supplementary material for: Association of exercise and ADHD symptoms: Analysis within an adult general population sample
Source: PLoS One. 2025 Feb 11;20(2):e0314508. doi: 10.1371/journal.pone.0314508 (PMC11813077; doi:10.1371/journal.pone.0314508)
Supplement: S1 Appendix — (DOCX) [file pone.0314508.s002.docx]

**S1 Appendix. Psychometric properties of measures used.**

**The Adult ADHD Self Report Scale – 6:** The scale has good concordance (*k*=.76), sensitivity (.69), and specificity (.99), and has established reliability for the sample targeted [67]. Within the current study, internal consistency was acceptable (Cronbach *α =* .69).

**The ADHD Rating Scale – IV with Adult prompts:** Excellent levels of internal reliability were found in the present study (inattentive section [items one-nine] *α*= .83; hyperactive section [items 10-18] *α* = .85; total [items 1-18] *α* = .89).

**The International Physical Activity Questionnaire** – **Long Format**, The scale has test-retest reliability clustered around .80, and criterion validity correlation of about .30, which matches with other self-report scales [69].
