# Supplementary material for: Association of exercise and ADHD symptoms: Analysis within an adult general population sample
Source: PLoS One. 2025 Feb 11;20(2):e0314508. doi: 10.1371/journal.pone.0314508 (PMC11813077; doi:10.1371/journal.pone.0314508)
Supplement: S2 Appendix — (DOCX) [file pone.0314508.s003.docx]

**S2 Appendix. Procedure and questionnaire.**

**Procedure**

Participants accessed the survey online via Qualtrics and were first presented with a detailed information sheet. They were then asked to indicate their consent via an electronic (Yes/No) check-box. Participants who did not consent to participate were directed to the end of the survey. Consenting participants were then asked to complete a series of demographic questions followed by the ASRS-6. After this, participants were presented with the questions on physical fitness, then questions on comorbid conditions and medication. Participants then were presented with the ADHD-RS-IV with Adult Prompts. Next, participants were presented with the IPAQ-L, depending on answers to certain questions, participants would skip other questions (as per the measures instructions). After that, participants were presented with the questions about what types of exercise they performed, use of fitness trackers and motivation for exercise. Finally, participants were presented with a debrief form. All survey sections required a response (except for the questions in the IPAQ-L a participant would have skipped), ensuring the survey was fully completed. There was no time limit, and after excluding outliers (defined as exceeding the 3^rd^ quartile of total duration + 3*IQR), average completion time was 14 minutes, 37 seconds.

**Questionnaire**

Start of Block: Information Page

**PARTICIPANT INFORMATION SHEET Version 1: 27/11/2020**
**Project Title:** Physical exercise: Links with attention, organisation and behavioural inhibition in adults 
You are being invited to take part in some research. Before you decide whether or not to participate, it is important for you to understand why the research is being conducted and what it will involve. Please read the following information carefully. 
What is the purpose of the research? We are conducting this research to explore how physical exercise may relate to aspects of our personality, mood and behaviour, such as our ability to pay/sustain attention, or control or inhibit our behaviour. We are seeking residents of the United Kingdom over 18 years of age to take part in this online study. Participation will take approximately 20 minutes.   Who is carrying out the research?  The data is being collected by Rory Tucker from the Department of Psychology in the College of Human and Health Sciences, under the supervision of Dr Claire Williams and Professor Phil Reed (Department of Psychology, College of Human and Health Sciences).  
What happens if I agree to take part? You will be asked to electronically provide your consent prior to taking part. If you consent to participate, we will ask you to provide some standard demographic details, such as your age, gender, employment status, and ethnicity. We will also you to record some other details, such as your weight and height (to the best of your knowledge) and how much sleep you typically get each night. After this, you will then be asked to complete a series of questionnaires about your behaviour, personality, and mood. For example, we will ask you to record how often you have performed certain physical activities (e.g., walking, running) and what types of exercise you have performed in the last 7 days. Similarly, we will ask you to rate aspects of your behaviour, such as whether you have been experiencing any difficulties paying attention, sitting still, organising your time, or finishing tasks. Typically, the questionnaires will ask you to indicate your level of agreement/disagreement to different statements and/or rate how well items describe you personally. Participation will take approximately 20 minutes. 
Are there any risks associated with taking part? The research has been approved by the College of Human and Health Sciences Research Ethics Committee. There are no significant risks associated with participation.  
Data Protection and Confidentiality Your data will be processed in accordance with the Data Protection Act 2018 and the General Data Protection Regulation 2016 (GDPR). All information collected about you will be kept strictly confidential. Your data will only be viewed by the research team.    All electronic data will be stored on a password-protected computer file on the Swansea University system, or on a password protected device owned by the research team.  Consent will be obtained electronically through Qualtrics and will contain no personally identifiable information.      Please note that all data collected will be anonymous from the point of participation. Thus, it will not be possible to identify and remove your data should you later decide to withdraw from the study. As the data is being collected online, once the data has been submitted you will be unable to withdraw your information. 
What will happen to the information I provide? An analysis of the information will form part of our report at the end of the study and may be presented to interested parties and published in scientific journals and related media.  Note that all information presented in any reports or publications will be anonymous and unidentifiable. 
Is participation voluntary and what if I wish to later withdraw? Your participation is entirely voluntary – you do not have to participate if you do not want to.  If you decide to participate, but wish to withdraw during the study, then you are free to withdraw at any time by closing the browser, without giving a reason and without penalty. Please note that removal of data will not be possible after the study has been completed, as all data will be anonymous from the start of participation. 
Data Protection Privacy Notice  The data controller for this project will be Swansea University. The University Data Protection Officer provides oversight of university activities involving the processing of personal data and can be contacted at the Vice Chancellors Office.  
Your personal data will be processed for the purposes outlined in this information page. Standard ethical procedures will involve you providing your consent to participate in this study by completing the consent form on the next page. 
The legal basis that we will rely on to process your personal data will be processing is necessary for the performance of a task carried out in the public interest. This public interest justification is approved by the College of Human and Health Sciences Research Ethics Committee, Swansea University. 
The legal basis that we will rely on to process special categories of data will be processing is necessary for archiving purposes in the public interest, scientific or historical research purposes or statistical purposes. 
How long will your information be held? Data will be preserved and accessible for a minimum of 10 years after completion of the research. Records from studies with major health, clinical, social, environmental or heritage importance, novel intervention, or studies which are on-going or controversial should be retained for at least 20 years after completion of the study. It may be appropriate to keep such study data permanently within the university, a national collection, or as required by the funder’s data policy.  
What are your rights? You have a right to access your personal information, to object to the processing of your personal information, to rectify, to erase, to restrict and to port your personal information. Please visit the University Data Protection webpages for further information in relation to your rights.  
Any requests or objections should be made in writing to the University Data Protection Officer:- 
University Compliance Officer (FOI/DP) Vice-Chancellor’s Office Swansea University Singleton Park Swansea SA2 8PP Email: dataprotection@swansea.ac.uk   
How to make a complaint If you are unhappy with the way in which your personal data has been processed you may in the first instance contact the University Data Protection Officer using the contact details above.  
If you remain dissatisfied then you have the right to apply directly to the Information Commissioner for a decision. The Information Commissioner can be contacted at: - 
Information Commissioner’s Office, Wycliffe House, Water Lane, Wilmslow, Cheshire, SK9 5AF www.ico.org.uk   
What if I have other questions? If you have further questions about this study, please do not hesitate to contact us: 
Researcher  Rory Tucker Department of Psychology Swansea University 907983@swansea.ac.uk 
Supervisor Professor Phil Reed Department of Psychology  Swansea University p.reed@swansea.ac.uk Supervisor Dr Claire Williams Department of Psychology Swansea University claire.williams@swansea.ac.uk 

End of Block: Information Page

Start of Block: Consent Form

**PARTICIPANT CONSENT FORM** **Version 1: 27/11/2020** **Project Title:** Physical exercise: Links with attention, organisation and behavioural inhibition in adults  Principle Researcher: Rory Tucker (907983@swansea.ac.uk)  Supervisors: Professor Phil Reed (p.reed@swansea.ac.uk); Dr Claire Williams (claire.williams@swansea.ac.uk)      1. I (the participant) confirm that I have read and understand the Participant Information sheet (version 1; dated 27/11/2020) for the above study, which was provided on the page before.  2. I understand that my participation is voluntary and that I am free to withdraw at any time during the study, without giving any reasons, but will not be able to withdraw my data after the questionnaire is completed.  3. I understand what my role will be in this research, and all my questions have been answered to my satisfaction.  4. I have been informed that the information I provide will be safeguarded.  5. I understand that I am free to ask any questions at any time before and during the study.  6. I agree to the researchers processing my personal data for any purposes connected with the research project as outlined to me.  7. I am happy for the information I provide to be used (anonymously) in academic papers and other formal research outputs.  8. I am aged 18 years or above.  9. I am a resident of the UK.

If you agree with all statements listed above, click **YES**. If you disagree with any of the statements above, click **NO** and you will be taken to the end of this survey. This study is being conducted by Swansea University, College of Human and Health Science.

- Yes
- No

*Thank you for your participation in this study!  Your help is very much appreciated*

End of Block: Consent Form

Start of Block: Demographic questions

| 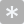 |
| --- |

**What is your age in years? (please use numbers as letters or spaces won't be processed)**

________________________________________________________________

**What is your gender?**

- Male
- Female
- Other
- Prefer not to say

**What is your ethnicity?**

- Welsh/English/Scottish/Northern Irish/British
- Irish
- Gypsy or Irish Traveller
- Any other White background
- White and Black Caribbean
- White and Black African
- White and Asian
- Any other Mixed / Multiple ethnic background
- Indian
- Pakistani
- Bangladeshi
- Chinese
- Any other Asian background
- African
- Caribbean
- Any other Black / African / Caribbean background
- Arab
- Other (Please enter) __________________________________________________

| 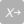 |
| --- |

**In which country do you currently reside?**

▼ United Kingdom ... Zimbabwe

**What is your highest level of education?**

- No formal education
- Secondary/Highschool (e.g. GCSE's) or equivalent
- College/Sixth form (e.g. BTEC, A-Levels) or equivalent
- University undergraduate (e.g. BSc, BA, Degree)
- Masters (e.g. MSC, MA)
- PhD/Doctorate
- Other (Please specify) __________________________________________________

**What is your employment status?**

- Full-time employment
- Part-time employment
- Unemployed as unable to work
- Currently unemployed but looking for work
- Retired
- Unpaid family work/Homemaker/Carer
- Full-time student
- Part-time student
- Unpaid voluntary work
- Other (Please specify) __________________________________________________

End of Block: Demographic questions

Start of Block: Demo qs

| 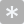 |
| --- |

**What is your age in years? (please use numbers as letters or spaces won't be processed)**

________________________________________________________________

**What is your gender?**

- Male
- Female
- Other
- Prefer not to say

**What is your ethnicity?**

- Welsh/English/Scottish/Northern Irish/British
- Irish
- Gypsy or Irish Traveller
- Any other White background
- White and Black Caribbean
- White and Black African
- White and Asian
- Any other Mixed / Multiple ethnic background
- Indian
- Pakistani
- Bangladeshi
- Chinese
- Any other Asian background
- African
- Caribbean
- Any other Black / African / Caribbean background
- Arab
- Other (Please enter) __________________________________________________

| 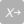 |
| --- |

**In which country do you currently reside?**

▼ United Kingdom ... Zimbabwe

**What is your highest level of education?**

- No formal education
- Secondary/Highschool (e.g. GCSE's) or equivalent
- College/Sixth form (e.g. BTEC, A-Levels) or equivalent
- University undergraduate (e.g. BSc, BA, Degree)
- Masters (e.g. MSC, MA)
- PhD/Doctorate
- Other (Please specify) __________________________________________________

**What is your employment status?**

- Full-time employment
- Part-time employment
- Unemployed as unable to work
- Currently unemployed but looking for work
- Retired
- Unpaid family work/Homemaker/Carer
- Full-time student
- Part-time student
- Unpaid voluntary work
- Other (Please specify) __________________________________________________

End of Block: Demo qs

Start of Block: ASRS - 6

| 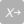 |
| --- |

**Check the box that best describes how you have felt and conducted yourself over the past 6 months.**

|  | Never | Rarely | Sometimes | Often | Very often |
| --- | --- | --- | --- | --- | --- |
| How often do you have trouble wrapping up the final details of a project, once the challenging parts have been done? |  |  |  |  |  |
| How often do you have difficulty getting things in order when you have to do a task that requires organization? |  |  |  |  |  |
| How often do you have problems remembering appointments or obligations? |  |  |  |  |  |
| When you have a task that requires a lot of thought, how often do you avoid or delay getting started? |  |  |  |  |  |
| How often do you fidget or squirm with your hands or feet when you have to sit down for a long time? |  |  |  |  |  |
| How often do you feel overly active and compelled to do things, like you were driven by a motor? |  |  |  |  |  |

End of Block: ASRS - 6

Start of Block: Physical fitness

**What is your height, in feet and inches to the best of your knowledge? E.g. enter 5'11" as "5" in Feet box and "11" in Inches box (1 foot = 30.5 cm, 1 inch = 2.5 cm)**
(please use numbers as letters or spaces won't be processed)

- Feet __________________________________________________
- Inches __________________________________________________

**What is your weight, in stone and pounds to the best of your knowledge? E.g. enter 12st 4Ibs as "12" in Stone box and "4" in Pounds box (1 stone = 6.35kg, 1 pound = 0.45kg)**
**(please use numbers as letters or spaces won't be processed)**

- Stone __________________________________________________
- Pounds __________________________________________________

**How many hours of sleep a day do you get on average?**

- 0-2
- 2-4
- 4-6
- 6-8
- 8-10
- 10+

End of Block: Physical fitness

Start of Block: Comorbidities

**Have you ever been diagnosed with any of the following conditions/disorders? Select any that apply**

- ⊗None
- A mood disorder (e.g Depression)
- Learning disabilities
- Substance use disorders
- ADHD (Attention Deficit Hyperactivity Disorder)
- Sleep disorders
- Anxiety disorders
- Conduct disorders
- Eating disorders
- Other (please specify) __________________________________________________

**If so, do you take any prescribed medication?**

- Yes (Please specify the disorder[s] you take it for) __________________________________________________
- No
- N/A (I do not have any of the above conditions/disorders)

End of Block: Comorbidities

Start of Block: Personality and mood

| 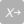 |
| --- |

**How well do the following statements describe your personality?**
 
I see myself as someone who....

|  | Disagree strongly | Disagree a little | Neither agree nor disagree | Agree a little | Agree strongly |
| --- | --- | --- | --- | --- | --- |
| is reserved |  |  |  |  |  |
| is generally trusting |  |  |  |  |  |
| tends to be lazy |  |  |  |  |  |
| is relaxed, handles stress well |  |  |  |  |  |
| has few artistic interests |  |  |  |  |  |
| is outgoing, sociable |  |  |  |  |  |
| tends to find fault with others |  |  |  |  |  |
| does a thorough job |  |  |  |  |  |
| gets nervous easily |  |  |  |  |  |
| has an active imagination |  |  |  |  |  |

| 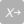 |
| --- |

**Over the last two weeks, how often have you been bothered by the following problems?**

|  | Not at all | Several days | More than half the days | Nearly every day |
| --- | --- | --- | --- | --- |
| Feeling nervous, anxious or on edge |  |  |  |  |
| Not being able to stop or control worrying |  |  |  |  |
| Feeling down, depressed or hopeless |  |  |  |  |
| Little interest or pleasure in doing things |  |  |  |  |

End of Block: Personality and mood

Start of Block: ADHD-RS-IV with adult prompts

| 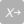 |
| --- |

**This questionnaire consists of 18 main items. Please read each main item (1-18) carefully and indicate the degree that each items affects you on the corresponding scale (None, Mild, Moderate, Severe).**
 
**Each main item (1-18) contains a number of prompts and questions, and you should select the highest applicable response on the scale (None, Mild, Moderate, or Severe) for each main item.**
 
**For example, if one prompt in Item 1 is scored "Moderate" and all the other prompts are "Mild", you should select “Moderate" as your overall response for Item 1.**

|  | None | Mild | Moderate | Severe |
| --- | --- | --- | --- | --- |
| 1. Do you make a lot of mistakes (in school or work)? Is this because you’re careless? Do you rush through work or activities? Do you have trouble with detailed work? Do you not check your work? Do people complain that you’re careless? Are you messy or sloppy? Is your desk or workspace so messy that you have difficulty finding things? |  |  |  |  |
| 2. Do you have trouble paying attention when watching movies, reading, or attending lectures? Or on fun activities such as sports or board games? Is it hard for you to keep your mind on school or work? Do you have unusual trouble staying focused on boring or repetitive tasks? Does it take a lot longer than it should to complete tasks because you can’t keep your mind on the task? Is it even harder for you than some others you know? Do you have trouble remembering what you read and do you need to re-read the same passage several times? |  |  |  |  |
| 3. Do people (spouse, boss, colleagues, friends) complain that you don’t seem to listen or respond (or daydream) when spoken to or when asked to do tasks? A lot? Do people have to repeat directions? Do you find that you miss the key parts of conversations because of drifting off in your own thoughts? Does it cause problems? |  |  |  |  |
| 4. Do you have trouble finishing things (such as work or chores)? Do you often leave things half done and start another project? Do you need consequences (such as deadlines) to finish? Do you have trouble following instructions (especially complex, multistep instructions that have to be done in a certain order with different steps)? Do you need to write down instructions, otherwise you will forget them? |  |  |  |  |
| 5. Do you have trouble organizing tasks into ordered steps? Is it hard prioritizing work and chores? Do you need others to plan for you? Do you have trouble with time management? Does it cause problems? Does difficulty in planning lead to procrastination and putting off tasks until the last moment possible? |  |  |  |  |
| 6. Do you avoid tasks (work, chores, reading, board games) that are challenging or lengthy because it’s hard to stay focused on these things for a long time? Do you have to force yourself to do these tasks? How hard is it? Do you procrastinate and put off tasks until the last moment possible? |  |  |  |  |
| 7. Do you lose things (eg, important work papers, keys, wallet, coats, etc)? A lot? More than others? Are you constantly looking for important items? Do you get into trouble for this (at work or at home)? Do you need to put items (eg, glasses, wallet, keys) in the same place each time, otherwise you will lose them? |  |  |  |  |
| 8. Are you ever very easily distracted by events around you such as noise (conversation, TV, radio), movement, or clutter? Do you need relative isolation to get work done? Can almost anything get your mind off of what you are doing, such as work, chores, or if you’re talking to someone? Is it hard to get back to a task once you stop? |  |  |  |  |
| 9. Do you forget a lot of things in your daily routine? Like what? Chores? Work? Appointments or obligations? Do you forget to bring things to work, such as work materials or assignments due that day? Do you need to write regular reminders to yourself to do most activities or tasks, otherwise you will forget? |  |  |  |  |
| 10. Can you sit still or are you always moving your hands or feet, or fidgeting in your chair? Do you tap your pencil or your feet? A lot? Do people notice? Do you regularly play with your hair or clothing? Do you consciously resist fidgeting or squirming? |  |  |  |  |
| 11. Do you have trouble staying in your seat? At work? In class? At home (eg, watching TV, eating dinner)? In church or temple? Do you choose to walk around rather than sit? Do you have to force yourself to remain seated? Is it difficult for you to sit through a long meeting or lecture? Do you try to avoid going to functions that require you to sit still for long periods of time? |  |  |  |  |
| 12. Are you physically restless? Do you feel restless inside? A lot? Do you feel more agitated when you cannot exercise on an almost daily basis? |  |  |  |  |
| 13. Do you have a hard time playing/working quietly? During leisure activity (nonstructured times or on your own such as reading a book, listening to music, playing a board game), are you agitated or dysphoric? Do you always need to be busy after work or while on vacation? |  |  |  |  |
| 14. Is it hard for you to slow down? Do you feel like you (often) have a lot of energy and that you always have to be moving, are always “on the go”? Do you feel like you’re driven by a motor? Do you feel unable to relax? |  |  |  |  |
| 15. Do you talk a lot? All the time? More than other people? Do people complain about your talking? Is it a problem? Are you often louder than the people you are talking to? |  |  |  |  |
| 16. Do you give answers to questions before someone finishes asking? Do you say things before it is your turn? Do you say things that don’t fit into the conversation? Do you do things without thinking? A lot? |  |  |  |  |
| 17. Is it hard for you to wait your turn (in conversation, in lines, while driving)? Are you frequently frustrated with delays? Does it cause problems? Do you put a great deal of effort into planning to not be in situations where you might have to wait? |  |  |  |  |
| 18. Do you talk when others are talking, without waiting until you are acknowledged? Do you butt into others’ conversations before being invited? Do you interrupt others’ activities? Is it hard for you to wait to get your point across in conversations or at meetings? |  |  |  |  |

End of Block: ADHD-RS-IV with adult prompts

Start of Block: IPAQ-L pt1

**You will now be presented with a sequence of questions for you to answer. Do not worry if the questions do not appear sequentially, simply answer the questions as presented to you**

**PART 1. 1) Do you currently have a job or do any unpaid work outside your home?**

- Yes
- No

Skip To: End of Block If PART 1. 1) Do you currently have a job or do any unpaid work outside your home?  = No

| 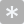 |
| --- |

**2) During the last 7 days, on how many days did you do vigorous physical activities like**
**heavy lifting, digging, heavy construction, or climbing up stairs as part of your work?**
**Think about only those physical activities that you did for at least 10 minutes at a time.**

- Days per week __________________________________________________
- No vigorous job-related physical activity

Skip To: Q9.5 If 2) During the last 7 days, on how many days did you do vigorous physical activities like heavy li... = No vigorous job-related physical activity

**3) How much time did you usually spend on one of those days doing vigorous physical activities as part of your work?**

- Hours per day: __________________________________________________
- Minutes per day: __________________________________________________

**4) Again, think about only those physical activities that you did for at least 10 minutes at a**
**time. During the last 7 days, on how many days did you do moderate physical activities**
**like carrying light loads as part of your work? Please do not include walking.**

- Days per week: __________________________________________________
- No moderate job-related physical activity

Skip To: Q9.7 If 4) Again, think about only those physical activities that you did for at least 10 minutes at a ti... = No moderate job-related physical activity

**5) How much time did you usually spend on one of those days doing moderate physical activities as part of your work?**

- Hours per day: __________________________________________________
- Minutes per day: __________________________________________________

**6) During the last 7 days, on how many days did you walk for at least 10 minutes at a time**
**as part of your work? Please do not count any walking you did to travel to or from**
**work.**

- Days per week: __________________________________________________
- No job-related walking

Skip To: End of Block If 6) During the last 7 days, on how many days did you walk for at least 10 minutes at a time as par... = No job-related walking

**7) How much time did you usually spend on one of those days walking as part of your work?**

- Hours per day __________________________________________________
- Minutes per day __________________________________________________

End of Block: IPAQ-L pt1

Start of Block: IPAQ-L pt2

**PART 2. 8) During the last 7 days, on how many days did you travel in a motor vehicle like a train, bus, car, or tram?**

- Days per week: __________________________________________________
- No traveling in a motor vehicle

Skip To: Q10.3 If PART 2. 8) During the last 7 days, on how many days did you travel in a motor vehicle like a trai... = No traveling in a motor vehicle

**9) How much time did you usually spend on one of those days traveling in a train, bus, car, tram, or other kind of motor vehicle?**

- Hours per day: __________________________________________________
- Minutes per day: __________________________________________________

**10) During the last 7 days, on how many days did you bicycle for at least 10 minutes at a time to go from place to place?**

- Days per week: __________________________________________________
- No bicycling from place to place

Skip To: Q10.5 If 10) During the last 7 days, on how many days did you bicycle for at least 10 minutes at a time to... = No bicycling from place to place

**11) How much time did you usually spend on one of those days to bicycle from place to place?**

- Hours per day: __________________________________________________
- Minutes per day: __________________________________________________

**12) During the last 7 days, on how many days did you walk for at least 10 minutes at a time to go from place to place?**

- Days per week: __________________________________________________
- No walking from place to place

Skip To: End of Block If 12) During the last 7 days, on how many days did you walk for at least 10 minutes at a time to go... = No walking from place to place

**13) How much time did you usually spend on one of those days walking from place to place?**

- Hours per day: __________________________________________________
- Minutes per day: __________________________________________________

End of Block: IPAQ-L pt2

Start of Block: IPAQ-L pt3

**PART 3. 14) Think about only those physical activities that you did for at least 10 minutes at a time. During the last 7 days, on how many days did you do vigorous physical activities like heavy lifting, chopping wood, shoveling snow, or digging in the garden or yard?**

- Days per week: __________________________________________________
- No vigorous activity in garden or yard

Skip To: Q11.3 If PART 3. 14) Think about only those physical activities that you did for at least 10 minutes at a... = No vigorous activity in garden or yard

**15) How much time did you usually spend on one of those days doing vigorous physical activities in the garden or yard?**

- Hours per day: __________________________________________________
- Minutes per day: __________________________________________________

**16) Again, think about only those physical activities that you did for at least 10 minutes at a time. During the last 7 days, on how many days did you do moderate activities like carrying light loads, sweeping, washing windows, and raking in the garden or yard?**

- Days per week: __________________________________________________
- No moderate activity in garden or yard

Skip To: Q11.5 If 16) Again, think about only those physical activities that you did for at least 10 minutes at a t... = No moderate activity in garden or yard

**17) How much time did you usually spend on one of those days doing moderate physical activities in the garden or yard?**

- Hours per day: __________________________________________________
- Minutes per day: __________________________________________________

**18) Once again, think about only those physical activities that you did for at least 10 minutes**
**at a time. During the last 7 days, on how many days did you do moderate activities like**
**carrying light loads, washing windows, scrubbing floors and sweeping inside your**
**home?**

- Days per week: __________________________________________________
- No moderate activity inside home

Skip To: End of Block If 18) Once again, think about only those physical activities that you did for at least 10 minutes a... = No moderate activity inside home

**19) How much time did you usually spend on one of those days doing moderate physical**
**activities inside your home?**

- Hours per day: __________________________________________________
- Minutes per day: __________________________________________________

End of Block: IPAQ-L pt3

Start of Block: IPAQ-L pt4

**PART 4. 20) Not counting any walking you have already mentioned, during the last 7 days, on how many days did you walk for at least 10 minutes at a time in your leisure time?**

- Days per week: __________________________________________________
- No walking in leisure time

Skip To: Q12.3 If PART 4. 20) Not counting any walking you have already mentioned, during the last 7 days, on how m... = No walking in leisure time

**21) How much time did you usually spend on one of those days walking in your leisure time?**

- Hours per day: __________________________________________________
- Minutes per day: __________________________________________________

**22) Think about only those physical activities that you did for at least 10 minutes at a time. During the last 7 days, on how many days did you do vigorous physical activities like aerobics, running, fast bicycling, or fast swimming in your leisure time?**

- Days per week: __________________________________________________
- No vigorous activity in leisure time

Skip To: Q12.5 If 22) Think about only those physical activities that you did for at least 10 minutes at a time. Du... = No vigorous activity in leisure time

**23) How much time did you usually spend on one of those days doing vigorous physical activities in your leisure time?**

- Hours per day: __________________________________________________
- Minutes per day: __________________________________________________

**24) Again, think about only those physical activities that you did for at least 10 minutes at a time. During the last 7 days, on how many days did you do moderate physical activities like bicycling at a regular pace, swimming at a regular pace, and doubles tennis in your leisure time?**

- Days per week: __________________________________________________
- No moderate activity in leisure time

Skip To: End of Block If 24) Again, think about only those physical activities that you did for at least 10 minutes at a t... = No moderate activity in leisure time

**25) How much time did you usually spend on one of those days doing moderate physical activities in your leisure time?**

- Hours per day: __________________________________________________
- Minutes per day: __________________________________________________

End of Block: IPAQ-L pt4

Start of Block: IPAG-L pt5

**PART 5: 26) During the last 7 days, how much time did you usually spend sitting on a weekday?**

- Hours per day: __________________________________________________
- Minutes per day: __________________________________________________

**27) During the last 7 days, how much time did you usually spend sitting on a weekend day?**

- Hours per day: __________________________________________________
- Minutes per day: __________________________________________________

End of Block: IPAG-L pt5

Start of Block: Exercise type

**In the last 7 days which forms of exercise listed here have you done? (select all that apply)**

- ⊗None
- Archery
- Basketball family (e.g. Basketball, Netball, etc)
- Bat sports (e.g. Cricket, Rounders, Baseball, etc)
- Bracket sports (e.g. Tennis, Badminton, etc)
- Climbing
- CrossFit
- Cycling
- Dancing
- Dodgeball
- Fencing
- Field events (Jumping) (e.g. Long jump, High jump, etc)
- Field events (Throwing) (e.g. shot put, Javelin, Frisbee, etc)
- Football
- Golf
- Gymnastics
- Hiking
- Hockey
- Horse riding
- Kayaking
- Martial Arts/Combat Sports (e.g. Karate, Wrestling, MMA, etc)
- Parkour/Free running
- Rowing
- Rugby
- Running
- Sailing
- Skating
- Skiing
- Snowboarding
- Surfing
- Swimming
- Trampolining
- Volleyball
- Walking
- Weight training
- Yoga
- Other (please specify) __________________________________________________

Carry Forward Selected Choices from "In the last 7 days which forms of exercise listed here have you done? (select all that apply)"

| 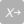 |
| --- |

**Please rank the forms of exercise you selected in order of which you have done the most in the last 7 days(e.g. 1- did this form of exercise the most in the last 7 days, 2 - did this form of exercise the second most in the last 7 days, etc). Do this by dragging the options up and down (with rank 1 at the top)**

⊗______ None

______ Archery

______ Basketball family (e.g. Basketball, Netball, etc)

______ Bat sports (e.g. Cricket, Rounders, Baseball, etc)

______ Bracket sports (e.g. Tennis, Badminton, etc)

______ Climbing

______ CrossFit

______ Cycling

______ Dancing

______ Dodgeball

______ Fencing

______ Field events (Jumping) (e.g. Long jump, High jump, etc)

______ Field events (Throwing) (e.g. shot put, Javelin, Frisbee, etc)

______ Football

______ Golf

______ Gymnastics

______ Hiking

______ Hockey

______ Horse riding

______ Kayaking

______ Martial Arts/Combat Sports (e.g. Karate, Wrestling, MMA, etc)

______ Parkour/Free running

______ Rowing

______ Rugby

______ Running

______ Sailing

______ Skating

______ Skiing

______ Snowboarding

______ Surfing

______ Swimming

______ Trampolining

______ Volleyball

______ Walking

______ Weight training

______ Yoga

______ Other (please specify)

**Do you use any fitness tracking tools (Fitbit, smartphone apps, etc)**

- Yes (Please specify ) __________________________________________________
- No

**What is your main reason / motivation for exercising? (if you do)**

- Fitness and health
- To lose weight
- For social reasons
- Self defence
- For my appearance
- Don't exercise
- Other (Please specify) __________________________________________________

End of Block: Exercise type

Start of Block: Debrief

**DEBRIEF FORM** **Version 1: 27/11/2020**    **Project Title: Physical exercise: Links with attention, organisation and behavioural inhibition**   Thank you for taking part in our research. Now that your contribution has finished, let us explain the rationale behind this work.     We are conducting this research to explore how physical exercise may relate to aspects of our personality, mood and behaviour, such as our ability to pay/sustain attention, or control or inhibit our behaviour. More specifically, we want to investigate whether physical exercise is related to Attention Deficit Hyperactive Disorder (ADHD) symptomology in adults.     Previous research has suggested that exercise may have beneficial effects on managing symptoms of ADHD, with exercise interventions also showing beneficial impacts on cognitive performance (e.g., improved working memory, concentration) and mood. However, previous studies have tended to focus on children with ADHD and there is currently insufficient data on this subject in adults. Our research seeks to address this gap and is why we asked you to answer questions about your exercise routine, physical activities, and behaviour.     As part of our research, we also asked you provide some standard demographic information (e.g., age, gender, employment status, and ethnicity), to rate your mood and personality characteristics, and to record some other details, including your weight and height and how much sleep you typically get each night.  This data will help us to determine whether any observed relationships between physical exercise and ADHD symptomology persist after controlling for these variables or act as mediators/moderators.     As a reminder, all information collected about you will be kept strictly confidential. Your data will only be viewed by the researcher/research team and will be stored for a minimum of 10 years after completion of the research.        An analysis of the information will form part of our report at the end of the study and may be presented to interested parties and published in scientific journals and related media. Please note that all information presented in any reports or publications will be anonymous and unidentifiable.     If you feel affected by issues raised by this research and would like to discuss any concerns, please contact the study Supervisors on the details provided below. If you feel this piece of research may have health implications for you, we advise you to contact your GP (family doctor) for initial support and information.      Further information about healthy lifestyles can also be found at https://www.nhs.uk/live-well/eat-well/     Further information and support for ADHD can be found at https://www.adhdfoundation.org.uk/information-support/     Swansea University Students can also contact the University Wellbeing services, for advice: Wellbeing Services, Horton Building, Swansea University, Singleton Park, Swansea, SA2 8PP, Tel: 01792 295592, www.swansea.ac.uk/wellbeing/        Researcher   Rory Tucker  Department of Psychology  Swansea University  907983@swansea.ac.uk    Supervisor  Professor Phil Reed  Department of Psychology   Swansea University  p.reed@swansea.ac.uk  Supervisor  Dr Claire Williams  Department of Psychology  Swansea University  claire.williams@swansea.ac.uk     For SurveyCircle users (www.surveycircle.com): The Survey Code is: ZD5D-PRYE-3PNJ-RSJW Please click the "next" button to submit results.

End of Block: Debrief
