# Supplementary material for: Association of exercise and ADHD symptoms: Analysis within an adult general population sample
Source: PLoS One. 2025 Feb 11;20(2):e0314508. doi: 10.1371/journal.pone.0314508 (PMC11813077; doi:10.1371/journal.pone.0314508)
Supplement: S3 Appendix — (DOCX) [file pone.0314508.s004.docx]

**S3 Appendix. Preliminary analysis of results**

Regarding demographic variables and their relationships with the two main variables (ADHD symptomology and PA level), no significant correlation was found between age and PA level (*r* = .097;  *p* = .114), but a significant negative correlation was found between age and ADHD symptomology (r = -.178; *p* = .003). There was no significant difference in PA level or ADHD symptomology based on gender (*U* = 6443, *p* =.915; *U* = 6408, *p* =.864; respectively), or education (*H*[4]= 3.7, *p* = .449; *H*[4] = 3.44, *p* = .488; respectively). There was no significant difference in ADHD symptomatology based on ethnicity (*H*[7] = 4.01, *p* = .779), or employment (*H* [2]= 1.87, *p* =.392), but PA level did differ significantly based on both ethnicity (*H*[7] = 22.01, *p* = .003) and employment (*H*[2] = 7.92, *p* =.019).
