# Supplementary figures and images for: Association of exercise and ADHD symptoms: Analysis within an adult general population sample
Source: PLoS One. 2025 Feb 11;20(2):e0314508. doi: 10.1371/journal.pone.0314508 (PMC11813077; doi:10.1371/journal.pone.0314508)

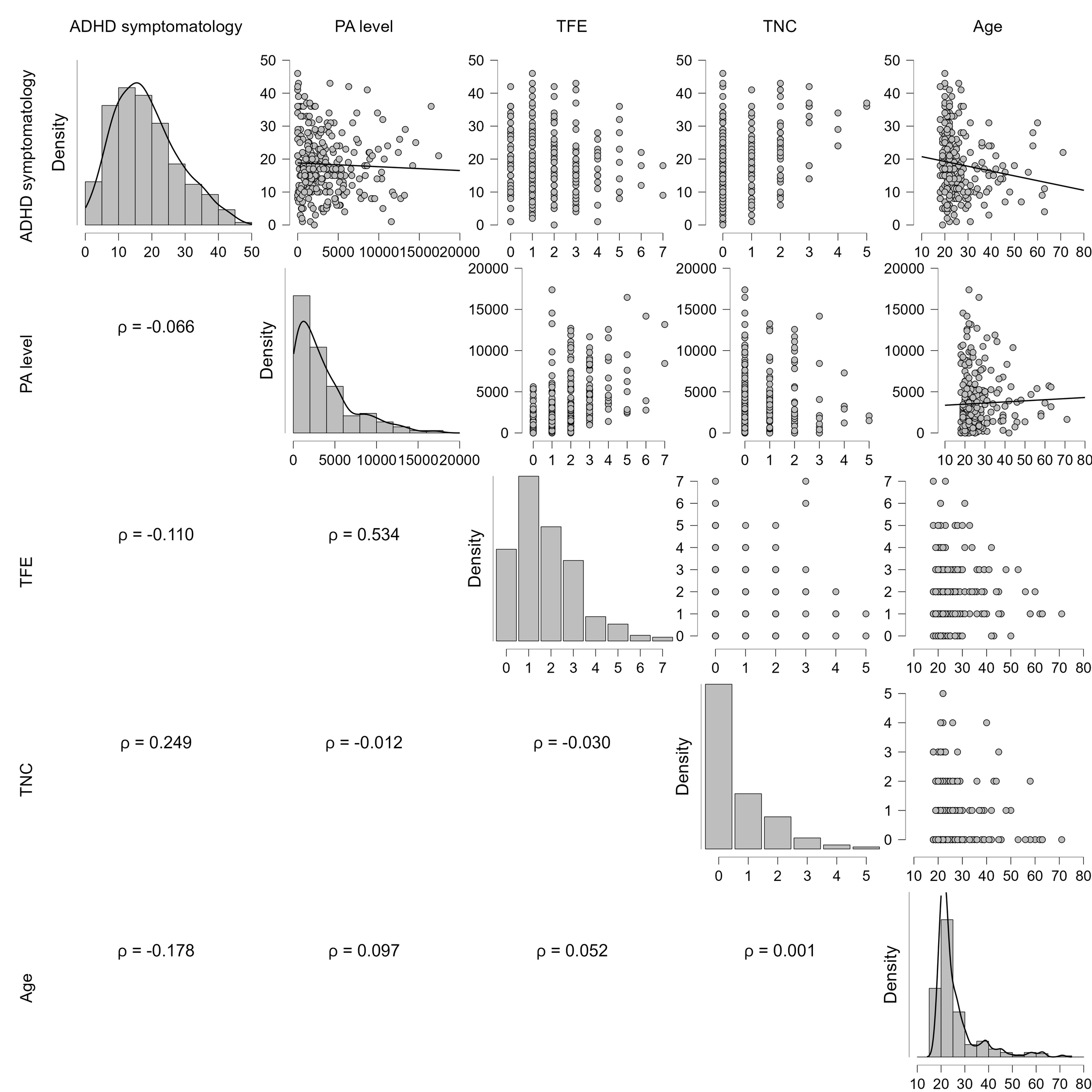

Supplement: S1 Fig — (TIF) [file pone.0314508.s005.tif]

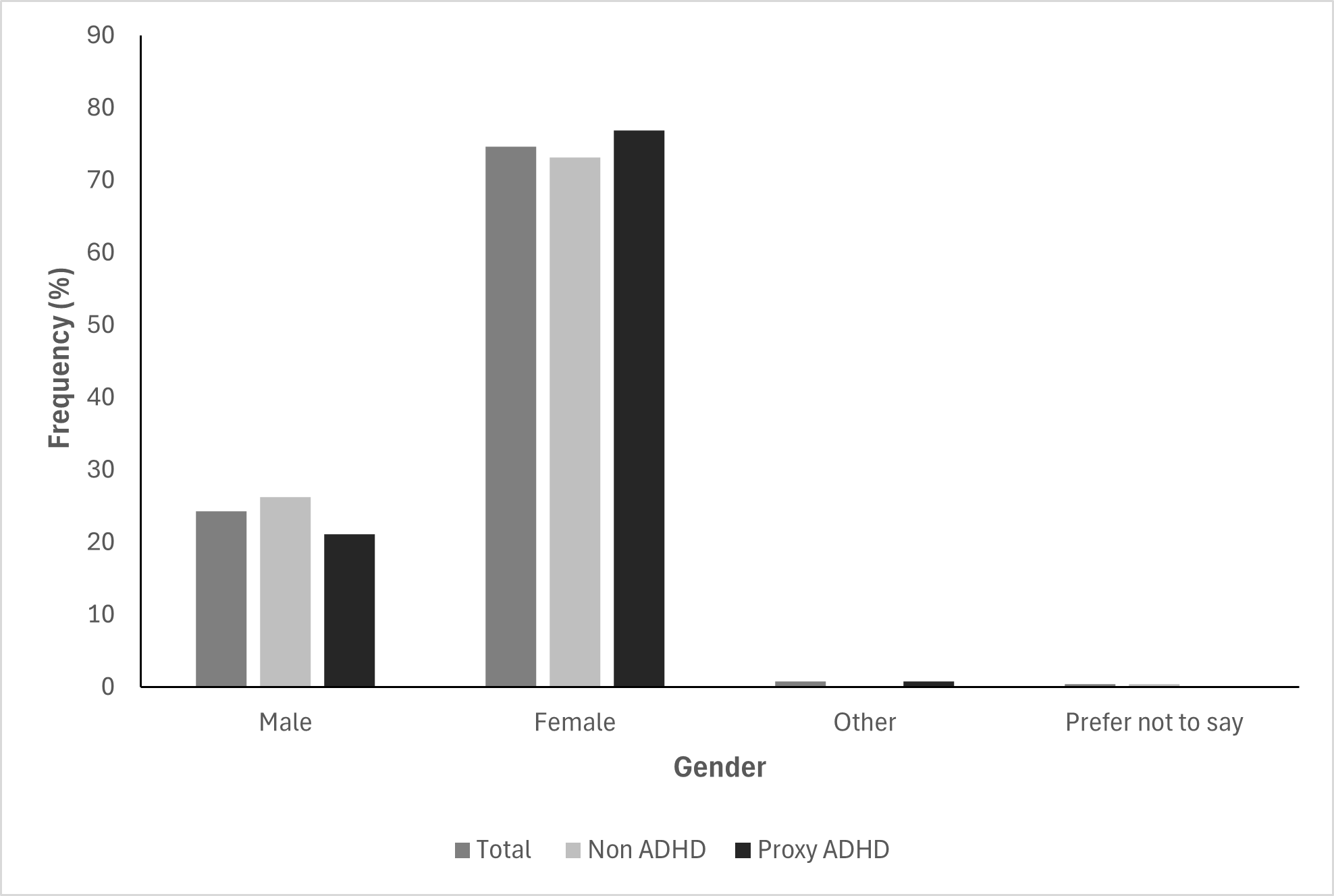

Supplement: S2 Fig — (TIF) [file pone.0314508.s006.tif]

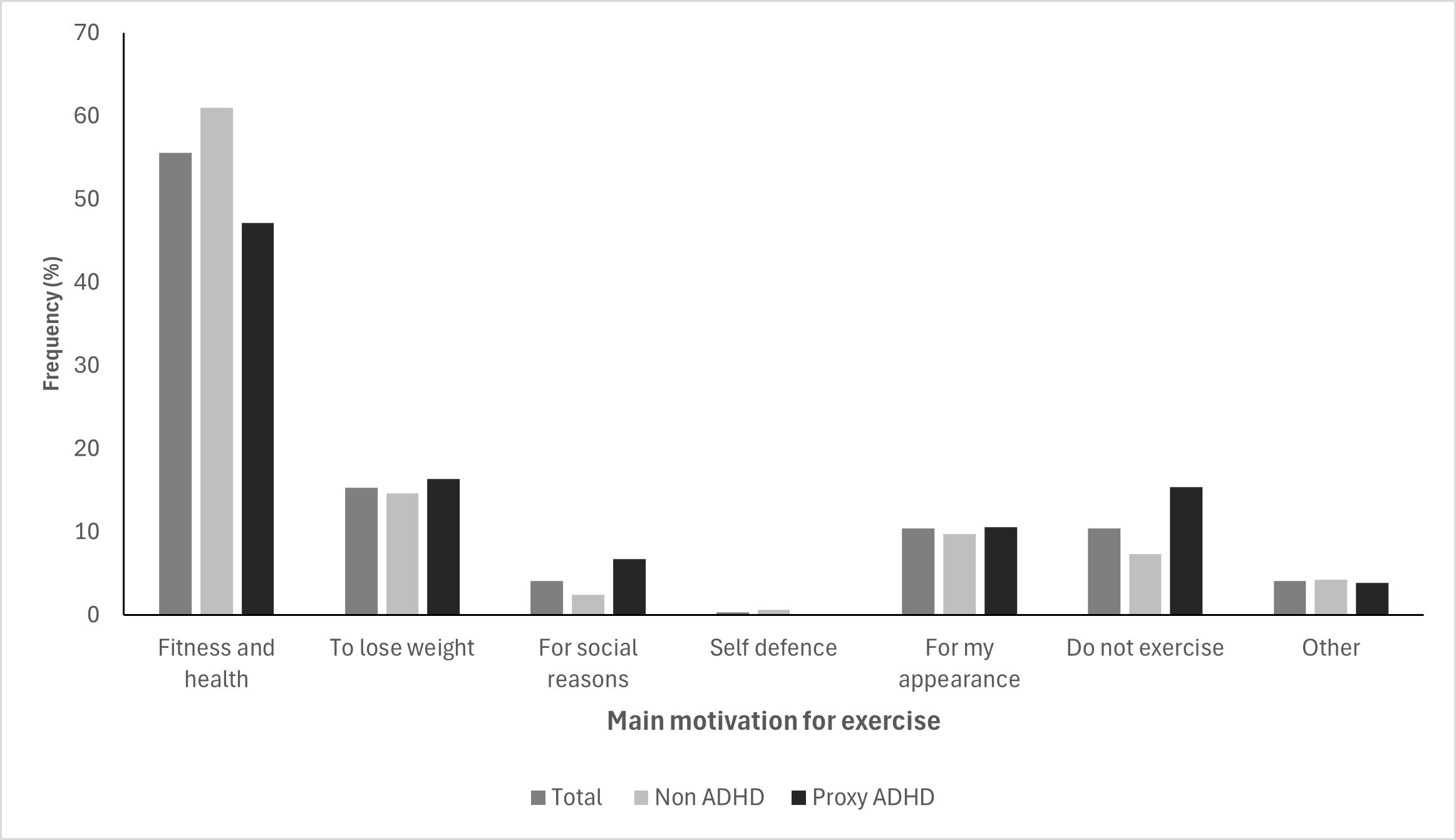

Supplement: S3 Fig — (TIF) [file pone.0314508.s007.tif]
