# Supplementary material for: Association of exercise and ADHD symptoms: Analysis within an adult general population sample
Source: PLoS One. 2025 Feb 11;20(2):e0314508. doi: 10.1371/journal.pone.0314508 (PMC11813077; doi:10.1371/journal.pone.0314508)
Supplement: S1 Table — (DOCX) [file pone.0314508.s008.docx]

**S1 Table.** **Demographic Information of the Study Sample**

| Demographics | n (%) |
| --- | --- |
| Gender |  |
| Male | 65 (24.25) |
| Female | 200 (74.63) |
| Other | 2 (0.75) |
| Prefer not to say | 1 (0.37) |
| Ethnicity |  |
| White/English/Scottish/Northern Irish/British | 167 (62.31) |
| Irish | 9 (3.36) |
| Any other white background | 41 (15.3) |
| White and black Caribbean | 3 (1.12) |
| White and Asian | 6 (2.24) |
| Any other Mixed/Multiple ethnic background | 3 (1.12) |
| Indian | 8 (2.99) |
| Pakistani | 5 (1.87) |
| Bangladeshi | 3 (1.12) |
| Chinese | 3 (1.12) |
| Any other Asian background | 7 (2.61) |
| African | 6 (2.24) |
| Caribbean | 2 (0.75) |
| Any other Black/African/Caribbean background | 2 (0.75) |
| Other | 3 (1.12) |
| Highest Level of education |  |
| No formal education | 1 (0.37) |
| Secondary/Highschool (e.g. GCSE’s) or equivalent | 6 (2.24) |
| College/Sixth form (e.g. BTEC, A-Levels) or equivalent | 68 (25.37) |
| University (e.g. BSc, BA, Degree) | 126 (47.01) |
| Masters (e.g. MSC, MA) | 53 (19.78) |
| PhD/Doctorate | 12 (4.48) |
| Other | 2 (0.75) |
| Employment Status |  |
| Full-time employment | 48 (17.91) |
| Part-time employment | 33 (12.21) |
| Unemployed as unable to work | 1 (0.27) |
| Currently unemployed but looking for work | 3 (1.11) |
| Retired | 1 (0.37) |
| Unpaid family work/Homemaker/Carer | 1 (0.37) |
| Full-time student | 172 (64.55) |
| Part-time student | 4 (1.49) |
| Unpaid voluntary work | 1 (0.37) |
| Other | 3 (1.12) |

Note: GCSE = General Certificate of Secondary Education; BTEC = business and Technology Education Council; A-level = Advanced level qualification; BSc = Bachelor of Sciences; BA = Bachelor of Arts; MSC = Master of Sciences; MA = Master of Arts; PhD = Doctorate of Philosophy.
