# Supplementary material for: Association of exercise and ADHD symptoms: Analysis within an adult general population sample
Source: PLoS One. 2025 Feb 11;20(2):e0314508. doi: 10.1371/journal.pone.0314508 (PMC11813077; doi:10.1371/journal.pone.0314508)
Supplement: S2 Table — (DOCX) [file pone.0314508.s009.docx]

**S2 Table. Descriptive Statistics and Correlation Coefficients (Spearman’s rho) for Study Variables (n=268)**

| Variable | Median | IQR | 1 | 2 | 3 | 4 | 5 |
| --- | --- | --- | --- | --- | --- | --- | --- |
| 1. ADHD symptomatology | 17 | 14 | - | - | - | - | - |
| 2. PA level | 2574.25 | 4031.25 | -0.66 | - | - | - | - |
| 3. TNC | 0 | 1 | .25** | -0.1 | - | - | - |
| 4. TFE | 1 | 2 | -.11 | .53** | -0.3 | - | - |
| 5. Mood | 4 | 5 | .41** | -.24** | .31** | -.22* | - |

*Note.* Statistical significance: **p* < .05; ***p* < .01. PA = Physical Activity; TNC = Total Number of Comorbidities; TFE = Total Forms of Exercise.
