# Supplementary material for: Association of exercise and ADHD symptoms: Analysis within an adult general population sample
Source: PLoS One. 2025 Feb 11;20(2):e0314508. doi: 10.1371/journal.pone.0314508 (PMC11813077; doi:10.1371/journal.pone.0314508)
Supplement: Attachment — Submitted filename: Review.pdf [file pone.0314508.s010.pdf]

Journal: PLOS One
Article: Association of exercise and ADHD symptoms: Analysis within an adult general population sample

Reviewer comments

The authors aimed to examine the the cross-sectional relationship between physical activity (PA) and attention deficit/hyperactivity disorder (ADHD) symptoms in a general population cohort acquired through convenience sampling. Studies such as this, that explore the role of behavioural factors in difficulties associated with mental health concerns, especially in relation to adult neurodevelopmental disorders are lacking. Such work is welcome as it leads to a deeper understanding of neurocognitive development and functioning and paves the way for the potential development of non-pharmacological interventions for those who wish to access such resources.

I have several comments and concerns that I would like to share with the authors below. I would like to apologise in advance if I have misinterpreted anything and I am open to clarification where needed.

Major Revisions:
Overall Structure and Expression

- There is the basis of a robust and important piece of scientific literature here, however extensive work is needed to improve the structure and expression of each section within. The paper at time deviates from the research questions posed at the start of the paper (e.g.BMI, Exploratory Variables section) and introduces important analyses and results at inappropriate times (e.g.BMI in Methods p 13 line 273; inattention and hyperactivity in the Discussion - p20 line 423)

Analysis performed and Presentation of Results

- The analyses performed in this paper are at times difficult to follow and new research questions are introduced in the methods and the results section (e.g. BMI, fitness tracking tools). Again, either the paper needs to be restructured to reflect these research questions or the sections need to be cut and the discussion re-written.
- The methods statistical analysis plan needs more detail. There are a number of analyses that aren't mentioned in the methods section but are then mentioned in the results and discussion. The most important is the analysis splitting of ADHD symptomology by sub-type. As highlighted in your introduction the dimensional assessment of ADHD presentation is an apparent strength of this work so I'm not sure why this section is in the supplementary material. I highly recommend included this research questions, analysis plan, and results in the main paper.
- Given the importance representation of females is given in the introduction there is little to no discussion of this in the paper.
- Mediation model looking at role of PA on the relationship between total forms of exercise (TFE) and ADHD Symptomology requires clarification around how this modelling was conducted and more extensive reporting of results is necessary.

Interpretation of results

- The discussion largely focusses on supplementary analyses that aren't mentioned in the main paper. It appears that these analyses epitomise elements of the aim of the paper - adopting a dimensional assessment of ADHD presentation, however they aren't at the forefront at all. For this reason, a complete restructure would be necessary to either include the statistical plan and results of these analyses in the main paper, or to re-frame the discussion in the context of the results reported.

Explanations and Minor revisions:

Introduction

- Although comprehensive at times, the introduction could be made a lot more concise. For example, the first 3 paragraphs could be condensed into a single paragraph that traverses ADHD definition, societal impact, and current intervention options..
- The paragraph that introduced PA is a times confusing due to the 'exercise' component. It could be a lot easier to read if everything is discussed in the context of PA (since exercise is a subset of it) and exercise is introduced when specifically referring to the literature.
- The paragraph on ADHD symptomology and PA needs extensive work. I suggest the following:
    - Discuss the literature that is directly relevant to your study - PA and ADHD symptoms in an adult population, not child studies.
    - There is almost an entire paragraph for Vysniauske et al 2020. Yes as an MA paper this is important, however as you have highlighted, the analysis includes children with ADHD and your work focuses on adults.
    - Please correct me if I wrong however as a reader we don't read about adults and ADHD until the end of paragraph 5 on pg 5 line 110. I suggest focussing on the 4 studies you have highlighted. These also need to be references
    - The focus does not need to be on what reviews have and have not covered as you are not writing a review.
- The paragraph on female representation in cohorts is good. We as the reader just need more on those 4 studies.
- The paragraph on dimensional assessment of ADHD is good but needs extensive re-working. I think what you are trying to say is that PA could help with specific elements of ADHD and a dichotomous diagnostic measure doesn't capture such variability? It becomes a bit confusing when the notion of 'undiagnosed individuals' is introduced and I am not 100% sure what you are trying to say sorry. However, this idea of dimensional assessment is extremely important and I encourage you to re-work this paragraph to confidently convey your point.
- The section on mediation could be condensed into a sentence or two, or potentially just included in the concluding paragraph as an aim.
- The concluding paragraph needs rewording. You have not specifically sampled adults with ADHD so I recommend caution in some of the wording that is introduced here and subsequently used throughout the paper.
    - "…relationship between level of PA and ADHD in adults" - this statement is not correct. It is ADHD symptoms in an adult general population cohort.

Methods

- The methods section appears to need re-working to improve the overall organisation and flow. It also appears to be missing some important analyses and includes results.
- I recommend starting with the Design subsection

Participants

- Further clarification is needed around inclusion and exclusion criterial and informed consent. I suggest it be included in the first paragraph of the participants section. Did exclusion criteria include the presence of other psychiatric conditions such as mood/personality disorders or neurological disorders?
- How did you define 'non-ADHD' as well as 'subclinical ADHD'. Was it anyone who didn't meet the ASRS-6 threshold? Report this grouping in the results section.
- A number or results are reported in the methods section.
    - Paragraph 2 line 162 onwards. This analyses needs to be stipulated in the statistical analysis section of the methods with the results reported in the results section. Both could be included in the supplementary material.
- In this section IPAQ-L is not defined (p7 line167).
- Need to define what constitutes an outlier (P5 line 169)
- Given an apparent strength of this paper is the representation of females, the statement on p8 line 171 should be written in the context of females not males.
- It's excellent to see such comprehensive demographic breakdown of the sample, however much of this can go in supplementary material.
- Table 1 needs to go in the results section. See more in results section of feedback

Measures

- Need a sentence at the start of each measure stating what purpose it serves e.g. "The ASRS-6 was used to classify the proxy diagnostic status (non ADHD and subclinical ADHD) of participants" rather than at the end.
- I strongly recommend not using 'subclinical diagnosis' for group and just using something like 'proxy diagnostic group' of which subclinical ADHD sits in. Because of this phrasing the paper at times it reads as if you are working with clinical participants, which you aren't.
- PHQ-4 and BFI-10 aren't references again in the main paper or provided supplementary material. If the measures aren't used please don't include them in the paper.
- Similarly analysis and results of BMI is discussed in the methods (p13 line 273) and not again in either the main paper or supplementary material. Please remove if not necessary. Otherwise define relevant research question.
- Information about scale psychometrics can go in supplementary material
- Procedure can largely go in supplementary material. This section also needs to include a copy of the questionnaire. Not enough information is provided in text about response options.
- Again need to define what outliers mean (p12 line 267)

Data Processing and Statistical Analysis

- Here I recommend a figure or sub-headings to help guide the reader on the different analyses that will be performed. These sub-headings can then be used in the results section.
- e.g. group classification, PA and ADHD Symptomology, Mediation of relationship between PA and ADHD Symptomology by group. This will then highlight the elements such as BMI and fitness trackers that don't really fit anywhere at this stage.

- Statistical analysis plan needs more detail. There are a number of analyses that aren't mention in the methods section but are then mentioned in the results and discussion. All analyses need to be explained in the methods and then reported in the results. This includes all preliminary testing such as correlations and t-tests as well as post-hoc testing.
- Need to add the following:
  - Mediation analyses
  - Inattention and Hyperactivity analyses
- BMI needs to be defined (pg 13 line 273)
- Again, measure descriptive statistics to be included in a table in results section, not methods.
- I recommend the visual depiction of models in the methods section

Results

- The results section is missing a number of analyses that are present in the supplementary materials and then referred to in the discussion. These need to be added to the main paper. I also recommend spending time making the results section more concise through better use of tables and visualisations, and where possible moving sections to the supplementary materials.
- The comments below reflect that that comments above would be addressed and the statistical analysis section of the methods would include all analyses performed.
- Much of the preliminary analyses can be moved to the supplementary materials. This of ways you can chunk the data to present in tables (e.g. H-tests)
- Table 1 needs to go in the results section and include information on all the key variables that are included in analysis. I recommend having 3 columns —> Total cohort and then the 2 groups (Proxy ADHD diagnostic grouping) :
  - Age
  - Gender
  - ADHD Symptomology
  - ASRS-6 Scores
  - ADHD-RS-IV Scores
  - IPAQ-L7S Scores
  - TNC
  - Table needs to include mean (SD) , min & max rather than median and IQR.
- Table notes needs to include definition of all acronyms. These days many people open tables and figures online so they need to be able to stand alone.
- Table 2.
  - Recommend moving to supplementary material.
  - These may be mistakes but it appears that a number of the correlation coefficients are quite large (e.g. 0.9) so it is suppressing that the p-values aren't significant.
  - There appears to be an incomplete row at the bottom of the table
  - Table is missing Age and I'm not sure why hours sleep is included when it isn't mentioned in the methods statistical analysis section
- Fig 1. Move to supplementary material. Big fan of this figure though!
- Table 3.
  - Remove group. See Table 1 comment for inclusion of grouping variable.

    - Motivation for exercise and fitness app use could be bar graphs or similar to help reader engagement, again split by group also.
  - Table 4.
    - Remove. See Table 1 comment for inclusion of grouping variable.
    - *Section 3.2 - Relationship between PA level and ADHD symptomology*
    - Overall this section is good.
    - Results table 5 needs to include the following
    - Definition of regression coefficients (B and beta) in notes
    - P-value in addition to staring
    - Consistency with terms - p 16 line 337 states comobidities rather than TNC
    - Could you please explain why there is a SE of 0 for the PA level as this indicates there is little to no variability.
- *Section 3.3 - Effect of Subclinical ADHD diagnosis on relationship between PA level and ADHD symptomology*
    - Overall a well written section, however I encourage the addition of a visual depiction of this model.
    - Again I strongly recommend not using ‘subclinical diagnosis’ for group and just using something like ‘proxy diagnostic group’ of which subclinical ADHD sits in. Because of this phrasing the paper at times it reads as if you are working with clinical participants, which you aren’t.
    - P18 line 372 ‘was’ instead of ‘is’; Please clarify what *b* represents here.
    - Table 6.
      - Please replace the term SCAD with something like ‘proxy diagnostic group’. Use consistent terminology throughout the paper.
      - Need to include the following
        - Standardised and unstandardised coefficient
        - Definition of regression coefficients (B and beta) in notes
        - ‘P’ and ’T’ need to be lower case and italicised
    - Fig 2 -
      - This figure could include more information in addition to the simple slopes
      - I recommend a scatter plot of all the data points and relevant slopes coloured by group with the group distributions for each variable on the axis. Screengrab example from Dipnall et al 2023 below:

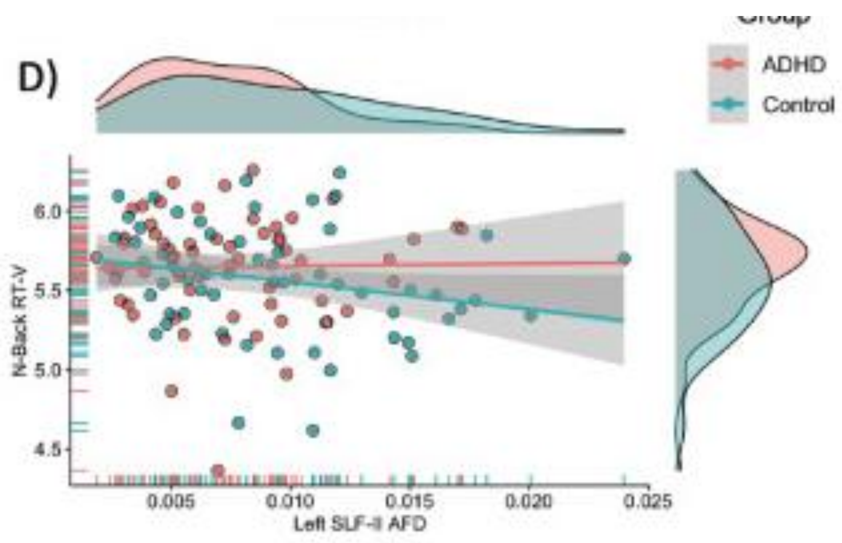

- *Section 3.4 Exploratory Variables*
    - These results were unexpected as they weren't discussed in the methods.
    - It isn't clear how use fitness tracking tools fits in with ADHD symptomology which is the primary outcome throughout the paper. Either this needs to be removed or further explanation and rationale is required.
    - I have some questions about the mediation analysis:
    - How was this analysis run?
    - Can you explain why PA accounted for 0.2% of variation of ADHD symptomology in section 3.4 but 0.1% of variation in section 3.2?
    - Is there a reason you did not control for age and TNC in the mediation model like what was done is section 3.2?
    - What were the direct and indirect effects of both PA and TFE on ADHD symptomology? I recommend showing this as a figure visualisation.
    - Need to discuss whether the 95% CIs crossed 0 (see Hayes, 2013)
- Table 7.
    - Definition of regression coefficients (B and beta) and acronyms in note needed
    - Addition of p-values
    - Need to include 95% CI for direct and indirect effects of PA and TFE

Supplementary Analyses

- These need to be added to the main paper with the analyses plan outlined in the methods in addition the results in the relevant section, not the discussion. Could you please provide more information on how these analyses were conducted.

Discussion

- In light of the feedback provided above I recommend the discussion is completely overhauled and re-worked. As it stands, the discussion largely focuses on analyses included in the supplementary materials.

References

- Please ensure references are formatted correctly (e.g. #40, #45, #61, #65)

Concluding Statement

The authors have the basis of an excellent paper and I hope these comments encourage, rather than dissuade the authors to continue building a stronger piece of work that will ultimately contribute more fully to the field. I look forward to hopefully seeing the next version.
