# Supplementary material for: Association of exercise and ADHD symptoms: Analysis within an adult general population sample
Source: PLoS One. 2025 Feb 11;20(2):e0314508. doi: 10.1371/journal.pone.0314508 (PMC11813077; doi:10.1371/journal.pone.0314508)
Supplement: Attachment — Submitted filename: REVIEW REPORT .pdf [file pone.0314508.s013.pdf]

Title: Association of exercise and ADHD symptoms: Analysis within an adult general population sample

This title is good and clearly states the independent and dependent variables.

Abstract

Though the abstract is good but the following need to be addressed

- One introductory statement (A brief introduction) is necessary to put the study into perspective. Just one sentence or two at most.
- In line 23-just as PA has been written in full, it would be good to first write ADHD in full then abbreviate later.

Introduction

- The introduction is clear and straight on point but in line 58-61 the authors have mentioned the challenges that arise as a result of ADHD but they have failed to mention the symptoms making the section unclear.
- The statement of the problem is clearly explained and is nicely linked to the purpose of the study.

Methodology

- The methodology is relevant, the inclusion and the exclusion criteria are quite clear.

Data Processing

- Line 266- Be specific which test statistic was used to analyze which data?

Descriptive statistics

2.5.1 – The authors should come out clear, is these inferential statistics or descriptive? This should be addressed.

- Line 339- is it conducing or conducting?

Analysis of results

- The authors used quantitative analyses and have clearly explained how they used each of the test statistic but what hypothesis was being tested? It is important for a quantitative study to have the hypothesis/ hypotheses stated.
- The findings that the study produces are very critical but have no implications, and they only need one or two sentences to bring out the implication of each finding.
- The manuscript is well written with key subtopics availed. The authors however need to be more interpretive in data presentation. The results are well analyzed and presented but have not been interpreted at all. Making it difficult to make exact meaning. It is always good practice to interpret all findings, this makes it easy to come up with the implications

of the findings. The manuscript would be more useful to a broader readership if the authors moved from just providing results to interpreting them and giving the implication as well.

- Generally, the whole section dealing with results needs further explanation and expansion.

DISCUSSION

The scope of the study is well provided and the presentation is consistent throughout the manuscript. The manuscript is well written and nicely presented quantitatively. This manuscript presents a balanced assessment of key constructs and shows evidence of being well researched, the following areas however need to be addressed;

- The discussion section should start with an objective or hypothesis of the study, this lays a good background for discussion.
- Avoid having recommendations in the discussion section.
- I tend to think that the authors oversimplified the findings thus inhibiting the deeper message that the subject under study could provide yet this is a very important study that can easily influence policy.
- If this study were to be replicated in a different population with similar parameters, would it yield similar/ consistent result? Authors should mention whether the study is generalizable and the gaps identified.
- Discussion should include current findings and relate it to past findings or similar studies elsewhere, this has not been done exhaustively.

Recommendations

- This section is good but should be refined to avoid repetition.

Conclusion

The conclusion is well written and is touching on the subject area. Secondly, conclusions are made based on the findings and discussion so avoid mixing this with recommendations, move all recommendations to the section designated for this. Authors should look at the aim of the study and conclude appropriately stating whether the objective(s) for the study was/ were achieved or not.

References

- These are well written

Minor Issues

- The study needs quick edit, there are a few mistakes on typos, tenses and spellings but generally, it is a good manuscript.
