# Supplementary material for: Association of exercise and ADHD symptoms: Analysis within an adult general population sample
Source: PLoS One. 2025 Feb 11;20(2):e0314508. doi: 10.1371/journal.pone.0314508 (PMC11813077; doi:10.1371/journal.pone.0314508)
Supplement: Attachment — Submitted filename: Response to Reviewers.docx [file pone.0314508.s011.docx]

**Response to Reviewers**

Dear Reviewers,

Thank you very much for your helpful and detailed feedback. We have responded to each of your suggestions and queries in the list below (organised by Reviewer Number), with our responses in red text. Please note that page and line numbers in our response correspond to the untracked version of the manuscript.

**Reviewer 1**

The article entitled “Association of exercise and ADHD symptoms: Analysis within an adult general population sample” explores an important topic regarding ADHD symptomatology which can lead to new approaches for ADHD management. However there are main issues which could be considered.

- The main conceptualization of the topic and its elaborations needs a major revision. The study is a descriptive cross sectional one which describes the relationship between physical activity and ADHD symptoms. However, the most parts of the introduction and discussion is devoted to the possible positive effects of physical activity “interventions” on ADHD symptoms. Such conclusion or attribution can not be obtained from the manuscript. The cross-sectional nature of the study is documented for the reader (see Page 6, Design). In the discussion section of the manuscript, we also explicitly recognise that future research should focus on investigating potential cause and effect relationships, highlighting in several places how (1) the correlational nature of our design means that cause and effect relationships cannot be established (e.g., pages 23, 26 and 27), and (2) that potential implications of study findings are speculative (e.g., Pages 26 and 27) as a result. With the latter, we offer alternative interpretations of main findings throughout and have generally amended our wording to be more speculative in nature also.

- Table 1 should be inserted in Results section. Abbreviations should be fully explained under the tables. In response to the comments from Reviewer 2, this table has been moved to the supplementary material under “S1 Table. Demographic Information of the Study Sample”. Abbreviations have been explained in the notes.

- It is mentioned that “The ADHD Rating Scale – IV with Adult prompts” has 9 subscales but only two of them was explained. We have amended our wording accordingly to bring additional clarity for the reader (see Page 10, line 222).

- PHQ-5 and The Big Five Inventory – 10 has been utilized in methodology but there are no data regarding these two questionnaires in the results. In line with thsi and other reviewer suggestions, we have since removed detailed mention of this questionnaire/inventory. For transparency though, it is still reported in brief as a potential moderator variable – but one that was not taken forward for further analysis (pg 10, line 236).

- Presentation of data in the results should be reordered. It should be initiated with demographic data following descriptive data and results of correlational analysis should be presented afterwards. Starting the result section with a table (Table 2) is not preferred. Moreover, table 2 is not clearly explained. On consideration of suggestions received from all reviewers, the results have been restructured so that they start with descriptive data (Tables 1 and 2 – with corresponding description in the main text body). Full demographic information for the sample has been moved to the supplementary information in response to feedback from Reviewer 2. What was Table 2 in the original manuscript has now been moved to supplementary material under “S2 Table. Descriptive Statistics and Correlation Coefficients (Spearman’s rho) for Study Variables (n=268)” - with further detail in the notes to explain it more clearly. Other correlations more central to the research aim are included further down in Table 3.

- Line 477-502 does not have relevancy with the main purpose of the manuscript We have considered the relevance of different material in response to reviewer feedback, including alignment between the aims, findings and discussion. Subsequently, the previous focus and discussion on fitness tracker usage (specifically lines 477 – 489 in the original manuscript) has been removed. However, material around motivation (lines 490-502 in the original manuscript, and lines 498-513 in the resubmitted manuscript) has been retained as we consider it relevant and important to the study - with reference to ‘motivation’ being incorporated into the introduction; aims and further parts of the discussion.

- Line 526-536: similar to what has been said in first comment, the conclusion you have mentioned cannot be speculated from your findings. As mentioned above, we have retained this section as we believe there is an important point to be made, even if speculative in nature at this stage. As mentioned previously though, we have amended our wording in several places to recognise possible alternative interpretations/explanations and the limitations attached to our correlational design.

**Reviewer 2**

**Major Revisions:**

*Overall Structure and Expression*

- There is the basis of a robust and important piece of scientific literature here, however extensive work is needed to improve the structure and expression of each section within. The paper at time deviates from the research questions posed at the start of the paper (e.g. BMI, Exploratory Variables section) and introduces important analyses and results at inappropriate times (e.g. BMI in Methods p 13 line 273; inattention and hyperactivity in the Discussion - p20 line 423). Thank you for your enthusiasm regarding the importance of this work. We have carefully considered the relevance of some variables - removing coverage of some that were less central and/or not analysed further (e.g., BMI; hours slept; mood; BFI; and fitness app use) whilst retaining reference to them as possible moderating variables. The analysis based on the splitting of ADHD symptomology by sub-type has also been moved from the supplementary material and incorporated into the main body, thus improving alignment between the results and discussion sections.

*Analysis performed and Presentation of Results*

- The analyses performed in this paper are at times difficult to follow and new research questions are introduced in the methods and the results section (e.g. BMI, fitness tracking tools). Again, either the paper needs to be restructured to reflect these research questions or the sections need to be cut and the discussion rewritten. The methods statistical analysis plan needs more detail. There are a number of analyses that aren’t mentioned in the methods section but are then mentioned in the results and discussion. The most important is the analysis splitting of ADHD symptomology by sub-type. As highlighted in your introduction the dimensional assessment of ADHD presentation is an apparent strength of this work so I’m not sure why this section is in the supplementary material. I highly recommend included

this research questions, analysis plan, and results in the main paper. As outlined previously, we have further considered the relevance and placement of more exploratory type variables – removing coverage of some that were less central and/or not analysed further (e.g., BMI; hours slept; mood; BFI; and fitness app use). As a requisite example, our previous discussion of fitness trackers as an exploratory variable has been removed from the Discussion section to enhance focus and alignment with the study aims. We have also included additional detail in the statistical analysis section, providing an overview of the approach taken for each core question/section of the results. Additionally, and given the importance of the dimensional assessment of ADHD, the analysis based on the splitting of ADHD symptomology by sub-type has been moved out of the supplementary material and incorporated into the main body as suggested.

- Given the importance representation of females is given in the introduction there is little to no discussion of this in the paper. Gender is discussed at various points, including on page 4/5 where we review the lack of significant difference in proxy diagnostic rates of ADHD when split by gender and offer possible explanations for this, and on Page 25, 27 and 28 we discuss the importance of ensuring representative samples in future research in this area.

- Mediation model looking at role of PA on the relationship between total forms of exercise (TFE) and ADHD Symptomology requires clarification around how this modelling was conducted and more extensive reporting of results is necessary. The intention of the exploratory multiple regression analysis was to control for PA rather than test its role as a mediator specifically. We have attempted to further clarify this component of the analysis in the results section.

*Interpretation of results*

- The discussion largely focusses on supplementary analyses that aren’t mentioned in the main paper. It appears that these analyses epitomise elements of the aim of the paper - adopting a dimensional assessment of ADHD presentation, however they aren’t at the forefront at all. For this reason, a complete restructure would be necessary to either include the statistical plan and results of these analyses in the main paper, or to re-frame the discussion in the context of the results reported. We have revised the discussion section considering your suggestions, removing discussion of less relevant and/or supplementary analyses (e.g., fitness tracker use) and streamlining other passages. As mentioned previously, and to improve alignment across sections and with the aims of the paper, we have incorporated the dimensional assessment of ADHD presentation analysis (previously in supplementary materials) to the main body of the paper. Given these changes, our discussion section is also more aligned – focussing on main analyses and findings reported within the manuscript body.

**Explanations and Minor revisions:**

**Introduction**

- Although comprehensive at times, the introduction could be made a lot more concise. For example, the first 3 paragraphs could be condensed into a single paragraph that traverses ADHD definition, societal impact, and current intervention Options. We have reviewed the introduction section generally and have streamlined the text in multiple places. As a specific example, the original three opening paragraphs have been condensed into one, covering ADHD definition, societal impact, and current intervention options as suggested.

- The paragraph that introduced PA is a times confusing due to the ‘exercise’ component. It could be a lot easier to read if everything is discussed in the context of PA (since exercise is a subset of it) and exercise is introduced when specifically referring to the literature. This paragraph has been revised in line with your suggestion – thank you (see Page 3, line 69).

- The paragraph on ADHD symptomology and PA needs extensive work. I suggest the

following:

- Discuss the literature that is directly relevant to your study - PA and ADHD symptoms in an adult population, not child studies. Discussion of the meta-analysis on children has been removed, with the studies now reported pertaining to adult samples (page 4, starting from line 77).
- There is almost an entire paragraph for Vysniauske et al 2020. Yes as an MA paper this is important, however as you have highlighted, the analysis includes children with ADHD and your work focuses on adults. This has now been removed.
- Please correct me if I wrong however as a reader we don’t read about adults and ADHD until the end of paragraph 5 on pg 5 line 110. I suggest focussing on the 4 studies you have highlighted. These also need to be references. Additional details (including corresponding citations/references) about the studies on adult samples has been included (page 4, starting from line 77).
- The focus does not need to be on what reviews have and have not covered as you are not writing a review. We have streamlined this section accordingly.

- The paragraph on female representation in cohorts is good. We as the reader just

need more on those 4 studies. Thank you for the positive feedback, and more details about those four studies can be found on Page 4, starting line 77.

- The paragraph on dimensional assessment of ADHD is good but needs extensive reworking. I think what you are trying to say is that PA could help with specific elements of ADHD and a dichotomous diagnostic measure doesn’t capture such variability? It becomes a bit confusing when the notion of ‘undiagnosed individuals’ is introduced and I am not 100% sure what you are trying to say sorry. However, this idea of dimensional assessment is extremely important and I encourage you to rework this paragraph to confidently convey your point. We have amended the corresponding paragraph to aid clarity (page 5, starting line 106).

- The section on mediation could be condensed into a sentence or two, or potentially just included in the concluding paragraph as an aim. This has been condensed.

- The concluding paragraph needs rewording. You have not specifically sampled adults with ADHD so I recommend caution in some of the wording that is introduced here and subsequently used throughout the paper. “…relationship between level of PA and ADHD in adults” - this statement is not correct. It is ADHD symptoms in an adult general population cohort. We have reworded this to read “ADHD *symptoms*” where applicable throughout the manuscript.

**Methods**

o The methods section appears to need re-working to improve the overall organisation and flow. It also appears to be missing some important analyses and includes results. I recommend starting with the Design subsection. We have reviewed the overall organisation and ordering of material within the methods section and have made several adjustments. We also open with the Design section as recommended (pg 6, line 142).

*Participants*

- Further clarification is needed around inclusion and exclusion criterial and informed consent. I suggest it be included in the first paragraph of the participants section. Did exclusion criteria include the presence of other psychiatric conditions such as mood/personality disorders or neurological disorders? Exclusion/inclusion criteria has been included (age, residence status in UK) and clarified in the first paragraph of the participants section (pg 7, starting line 150). Information about informed consent has also been added.

- How did you define ‘non-ADHD’ as well as ’subclinical ADHD’. Was it anyone who didn’t meet the ASRS-6 threshold? Report this grouping in the results section. We have clarified our approach at the start of the Data Processing and statistical analysis subsection (page 11, line 262), as well as in the description of the ASRS-R measure (page 9, line 206).

- A number or results are reported in the methods section. All results now appear in the results or supplementary materials, as applicable and based on your recommendations.

- Paragraph 2 line 162 onwards. This analyses needs to be stipulated in the statistical analysis section of the methods with the results reported in the results section. Both could be included in the supplementary material. For transparency and ease of access, we have opted to retain this detail within the main body of the paper. As it pertains to pre-processing of data (i.e., data exclusion rules) rather than statistical analysis per se, we feel that it is appropriately placed within its current section and so that the final sample is established at that point.

- In this section IPAQ-L is not defined (p7 line167). This has been corrected.

- Need to define what constitutes an outlier (P5 line 169). Thank you for identifying this oversight. This has now been corrected (pg 7, line 165).

- Given an apparent strength of this paper is the representation of females, the statement on p8 line 171 should be written in the context of females not males. Amended accordingly.

- It’s excellent to see such comprehensive demographic breakdown of the sample, however much of this can go in supplementary material. Changed to be included as supplementary information under “S1 Table. Demographic Information of the Study Sample”

- Table 1 needs to go in the results section. See more in results section of feedback. Table 1 (demographics) has been moved to the supplementary material as advised above.

*Measures*

o Need a sentence at the start of each measure stating what purpose it serves e.g. “The ASRS-6 was used to classify the proxy diagnostic status (non ADHD and subclinical ADHD) of participants” rather than at the end. This has been actioned (page 9/10) (e.g., the ASRS-6 was used to classify the proxy diagnostic status of participants in the current study; The IPAQ-L was used to measure PA Level).

o I strongly recommend not using ‘subclinical diagnosis’ for group and just using something like ‘proxy diagnostic group’ of which subclinical ADHD sits in. Because of this phrasing the paper at times it reads as if you are working with clinical participants, which you aren’t. Thank you for this helpful suggestion. We have amended to ‘proxy diagnostic group’ as recommended.

o PHQ-4 and BFI-10 aren’t references again in the main paper or provided supplementary material. If the measures aren’t used please don’t include them in the paper. We have removed detailed mention of these but for transparency, we retain mention of them as potential moderator variables that were included in the initial survey but not taken forward to further analysis (pg 10, line 236).

o Similarly analysis and results of BMI is discussed in the methods (p13 line 273) and not again in either the main paper or supplementary material. Please remove if not necessary. Otherwise define relevant research question. Same as above, we have removed but mentioned the variable as a potential moderator variable that was included in the initial survey but not analysed further.

o Information about scale psychometrics can go in supplementary material Changed to be included as supplementary information under “S1 Appendix. Psychometric properties of measures used.”

o Procedure can largely go in supplementary material. This section also needs to include a copy of the questionnaire. Not enough information is provided in text about response options. Changed to be included as supplementary information under “S2 Appendix. Procedure and questionnaire” - including a copy of the full survey for readers to review.

o Again need to define what outliers mean (p12 line 267). This has been corrected to include the relevant details (pg 11, line 252).

*Data Processing and Statistical Analysis*

o Here I recommend a figure or sub-headings to help guide the reader on the different

analyses that will be performed. These sub-headings can then be used in the results

section. e.g. group classification, PA and ADHD Symptomology, Mediation of relationship

between PA and ADHD Symptomology by group. This will then highlight the elements

such as BMI and fitness trackers that don’t really fit anywhere at this stage. - Corrected in line with your suggestion.

o Statistical analysis plan needs more detail. There are a number of analyses that aren’t mention in the methods section but are then mentioned in the results and discussion. All analyses need to be explained in the methods and then reported in the results. This includes all preliminary testing such as correlations and t-tests as well as post-hoc testing. Corrected except for t-tests and post hoc tests as these were not included.

o Need to add the following:

o Mediation analyses. We are uncertain about what is meant by this as a mediation analysis was not performed. This might refer to either the moderation analysis or the multiple regression at the end, both of which have been clarified further though.

o Inattention and Hyperactivity analyses Corrected

o BMI needs to be defined (pg 13 line 273). This analysis has subsequently been removed based on your other feedback and recommendations elsewhere.

o Again, measure descriptive statistics to be included in a table in results section, not

methods. Corrected (see current Tables 1 [pg 14] and 2 [pg 15] in results).

o I recommend the visual depiction of models in the methods section A model of the main moderation analysis has been included as Figure 1 (pg 12, Line 290).

**Results**

o The results section is missing a number of analyses that are present in the supplementary materials and then referred to in the discussion. These need to be added to the main paper. I also recommend spending time making the results section more concise through better use of tables and visualisations, and where possible moving sections to the supplementary materials. Thank you for your helpful suggestions. We have made a number of changes throughout the results section, including movement of one main analysis from the supplementary material to the main body, removal of other less relevant and/or exploratory analyses, and have added visualisations (e.g., Figure 2 [pg 19, line 394]).

o The comments below reflect that that comments above would be addressed and the

statistical analysis section of the methods would include all analyses performed.

o Much of the preliminary analyses can be moved to the supplementary materials. This

of ways you can chunk the data to present in tables (e.g. H-tests) Changed to be included as supplementary information under “S3 Appendix. Preliminary analysis of results.”

o Table 1 needs to go in the results section and include information on all the key

variables that are included in analysis. I recommend having 3 columns —> Total

cohort and then the 2 groups (Proxy ADHD diagnostic grouping) : The original Table 1 (demographic information of the study sample) has been moved to the supplementary materials. Table 1 presented in the results section (pg 14) now presents the descriptive statistics for the continuous study variables (split across three columns as suggested) and Table 2 (pg 15) presents the frequencies for gender and main motivation for exercise (similarly split into three columns).

o Age

o Gender

o ADHD Symptomology

o ASRS-6 Scores

o ADHD-RS-IV Scores

o IPAQ-L7S Scores

o TNC

o Table needs to include mean (SD) , min & max rather than median and IQR. This has been actioned.

o Table notes needs to include definition of all acronyms. These days many people open tables and figures online so they need to be able to stand alone. Corrected.

o Table 2. Recommend moving to supplementary material. Changed to be included as supplementary information under “S2 Table. Descriptive Statistics and Correlation Coefficients (Spearman’s rho) for Study Variables (n=268)”

These may be mistakes but it appears that a number of the correlation coefficients are quite large (e.g. 0.9) so it is suppressing that the p-values aren’t significant. No longer applicable as “Hours slept” is no longer used or reported in the main analysis. However, all remaining values have also been checked for accuracy.

There appears to be an incomplete row at the bottom of the table. This has been corrected (see S2 Table. Descriptives Statistics and Correlation Coefficients...).

Table is missing Age and I’m not sure why hours sleep is included when it isn’t mentioned in the methods statistical analysis section Correlations with age are included under S3 Appendix. Hours slept variable has been removed.

Fig 1. Move to supplementary material. Big fan of this figure though! Thank you. Changed to be included as supplementary information under “S1 Figure. Correlations matrix of scatterplots and histograms for ADHD symptomatology, PA level; Mood; TFE; TNC and Age”

Table 3: (1) Remove group. See Table 1 comment for inclusion of grouping variable. Corrected, and (2) Motivation for exercise and fitness app use could be bar graphs or similar to help reader engagement, again split by group also. Table is split by group and bar graphs are included as supplementary information under “S2 Figure. Frequencies of gender across Proxy diagnostic groups” and “S3 Figure. Frequencies of Main motivation for exercise across Proxy diagnostic groups”

o Table 4. Remove. See Table 1 comment for inclusion of grouping variable. This has been removed.

*Section 3.2 - Relationship between PA level and ADHD symptomology*

o Overall this section is good. Thank you for your comments.

o Results table 5 needs to include the following

o Definition of regression coefficients (B and beta) in notes Corrected

o P-value in addition to staring Corrected

o Consistency with terms - p 16 line 337 states comorbidities rather than TNC. Corrected

- Could you please explain why there is a SE of 0 for the PA level as this indicates there is little to no variability. Output tables have been triple checked for accuracy – with valued determined to be correctly reported. SE was put as “<.01” to show that it wasn’t exactly 0.

*Section 3.3 - Effect of Subclinical ADHD diagnosis on relationship between PA level and*

*ADHD symptomology*

o Overall a well written section, however I encourage the addition of a visual depiction of this model. Visual model included as Figure 1 in the data processing and statistical analysis subsection (pg 12, line 290). Thank you for the suggestion of the figure which we feel has strengthened the reporting of those findings.

o Again I strongly recommend not using ‘subclinical diagnosis’ for group and just using something like ‘proxy diagnostic group’ of which subclinical ADHD sits in. Because of this phrasing the paper at times it reads as if you are working with clinical participants, which you aren’t. Corrected. We have adopted ‘proxy diagnostic group’ throughout.

o P18 line 372 ‘was’ instead of ‘is’; Please clarify what b represents here. Corrected

o Table 6.

▪ Please replace the term SCAD with something like ‘proxy diagnostic

group’. Use consistent terminology throughout the paper. Corrected.

▪ Need to include the following

• Standardised and unstandardised coefficient Unstandardised coefficients were included, but standardised coefficients have not been included in line with the recommendations by Hayes

• Definition of regression coefficients (B and beta) in notes Corrected

• ‘P’ and ’T’ need to be lower case and italicised Corrected

o Fig 2 -

▪ This figure could include more information in addition to the simple

slopes

▪ I recommend a scatter plot of all the data points and relevant slopes

coloured by group with the group distributions for each variable on

the axis. Screengrab example from Dipnall et al 2023 below: Corrected – see Figure 2 (pg 19, line 394).

*Section 3.4 Exploratory Variables*

o These results were unexpected as they weren’t discussed in the methods. These tests have now been discussed more clearly in the Data processing and statistical analysis subsection of the methods (pg 12 and 13).

o It isn’t clear how use fitness tracking tools fits in with ADHD symptomology which is the primary outcome throughout the paper. Either this needs to be removed or further explanation and rationale is required. This has now been removed.

o I have some questions about the mediation analysis: We have further clarified the regression analysis, where the intention was to control for PA rather than test its role as a mediator specifically.

o How was this analysis run? See previous comment.

o Can you explain why PA accounted for 0.2% of variation of ADHD symptomology in section 3.4 but 0.1% of variation in section 3.2? This only referred to the *additional* amount of variance PA added to the already existing model, not the total amount (and therefore would differ when entered in different steps/models).

o Is there a reason you did not control for age and TNC in the mediation model like what was done is section 3.2? It was not deemed necessary as this was only an exploratory analysis and controlling for the variables “age” and “TNC” was not predicted to contribute as much to this analysis as it would with the central analysis in section 3.2.

o What were the direct and indirect effects of both PA and TFE on ADHD

symptomology? I recommend showing this as a figure visualisation. As mentioned above, this was a multiple linear regression, not a mediation analysis and therefore direct/indirect effects are not applicable in this instance.

o Need to discuss whether the 95% CIs crossed 0 (see Hayes, 2013) 95% CIs is included

o Table 7.

o Definition of regression coefficients (B and beta) and acronyms in note needed Corrected

o Addition of p-values Corrected

o Need to include 95% CI for direct and indirect effects of PA and TFE. As mentioned above, direct/indirect effects were not considered applicable, however 95% CI were included for unstandardised regression weights.

**Supplementary Analyses**

o These need to be added to the main paper with the analyses plan outlined in the methods in addition the results in the relevant section, not the discussion. Could you please provide more information on how these analyses were conducted. As explained previously, we have moved some core analysis from the supplementary material to the main body (results), explained the approach to analysis in greater detail in the methods section (statistical analysis), and have removed mention of additional and more exploratory analyses from the discussion section (e.g., fitness tracker).

**Discussion**

o In light of the feedback provided above I recommend the discussion is completely

overhauled and re-worked. As it stands, the discussion largely focuses on analyses

included in the supplementary materials. Supplementary material has been reworked and integrated into the main paper and the discussion has been reformatted to reflect this. Additionally, sections of the discussion pertaining to aspects that have now been removed from the main paper (e.g., fitness app use) have likewise been removed from the discussion.

**References**

- Please ensure references are formatted correctly (e.g. #40, #45, #61, #65) Corrected

**Reviewer 3**

**Introduction**

You should add more references/studies on the psychiatrics/clinical aspects. You mentioned very well how ADHD can affect cost, but need be more specified how aggressive can be this disorder, often is underestimated compared to other psychiatric disorders. Add also something about the comorbidities and suicide risk. In line with comments from Reviewer 2, the opening paragraphs of the introduction section have been streamlined, but we have added reference to comorbidities and suicide risk (lines 55-57) – as well as retaining discussion of how ADHD can affect cost.

- Row 29-30: In contrast to research with children, please reformulate that. This has been reworded (see lines 30,31).

- Row 35: non-specific variables/ measures are used. Give an example. This has been included (see lines 38,39).

- Row 56-57: sum up cost, you named it two times. Amended accordingly (see lines 57,58).

- Row 82: improve cognitive function, name the most important. This has been included (e.g., planning, scheduling, working memory, task coordination) (see line 72).

- Row 88: cognitive deficit, name the most important. This has been included (see lines 78,79).

- Row 145: a further aim was. I think you need a future form. We have reviewed the final paragraph carefully and have ensured that the tenses/wording have been phrased consistently across that section (all other mention of aims have used the same tense).

**Discussion**

- Row 537 - 539 : reformulate the sentence. This has been reworded (see lines 551-553).

- Row 595- 599 : reformulate the sentence, maybe create two different sentences. This has been reworded (see lines 613-616).
