# Supplementary material for: Association of exercise and ADHD symptoms: Analysis within an adult general population sample
Source: PLoS One. 2025 Feb 11;20(2):e0314508. doi: 10.1371/journal.pone.0314508 (PMC11813077; doi:10.1371/journal.pone.0314508)
Supplement: Attachment — Submitted filename: Response to Reviewers.docx [file pone.0314508.s012.docx]

**Response to Reviewers**

Dear Reviewers,

Thank you very much for your feedback. We have responded to each of your suggestions and queries in the list below (organised by Reviewer Number), with our responses in red text. Please note that page and line numbers in our response correspond to the *untracked* version of the manuscript.

**Reviewer #2**

Thank you for addressing my extensive comments and concerns. Well done on producing a strong piece of work. Thank you very much for your feedback and detailed comments that have allowed us to improve our work.

**Reviewer #3**

- 55-57: "along with poor attention and memory, to the point that it interferes with development or functioning [1]." Here I would like you to be more specific on cognitive deficits, is not only memory and attention involved in this disorder. Furthermore, not all memory domains is compromised but some memory aspects/functions. Executive functions are also very important and nevertheless social cognition which is very little named for this disorder. Add some part of cognition and few references. Thank you for this suggestion. We have included further information and specificity concerning some of the cognitive deficits associated with ADHD (e.g., aspects of memory, executive functions). We have also included information about social cognitive deficits as suggested (see lines___).
- 59: please spell NHS. Thank you for highlighting this oversight. We have now defined NHS on first mention (see line 65).
- 89-96: try to sum it up. We have inserted an additional sentence at the end of this paragraph, summarising the key take home message from the literature reviewed (see lines 102-104).
- 110: symptomology (the point is missing). A period (full stop) has been inserted (see line 118).
- 238: average hours slept; and fitness app use. Add References. The measures for hours slept and fitness app use were devised for the purposes of this study – which has now been further clarified in the manuscript (see line 246). We have also removed reference to ‘standardised’ in the sub-section title (see line 243).
- 306: table 1 Group Total (n= 268) Non-ADHD (n=164) Proxy Diagnostic (n=104) ---> between group and next raw there is no space. Visually do not look very clear maybe write GROUP in capitals or add a space or a line. Same comments for table 2. A line has been added (table boundary) below Sample/Group (Tables 1 and 2 – pages 14 and 15) to aid clarity. We have also amended Group to ‘Sample/Group’ to make it clearer when data corresponds to the total sample versus the ADHD groups. Please note that Microsoft word does not track changes regarding table boundaries so this change will not be displayed in the document with tracked changes.
- 428-436 and 439: in the discussion, add references in the text of previous similar findings. References have been added to the first paragraph of the discussion, highlighting findings of previous research (see lines 439-447).
- 610-613: reformulate. and also clarify the role of confounders. The section has been reworded to aid clarity and includes additional information regarding the role of confounders (See lines 617-629).
