# Supplementary material for: Association of exercise and ADHD symptoms: Analysis within an adult general population sample
Source: PLoS One. 2025 Feb 11;20(2):e0314508. doi: 10.1371/journal.pone.0314508 (PMC11813077; doi:10.1371/journal.pone.0314508)
Supplement: Attachment — Submitted filename: Response to Reviewers.docx [file pone.0314508.s014.docx]

**Response to Reviewers**

Dear Reviewers,

Thank you very much for your feedback. We have responded to each of your suggestions and queries in the list below (organised by Reviewer Number), with our responses in red text. Please note that page and line numbers in our response correspond to the *untracked* version of the manuscript.

**Reviewer #4**

Based on previous reviewers ' recommendations, the article has undergone considerable editing and restructuring of the main concepts and methodology. For future studies/articles, it would behove the authors to carry out more thorough preparation and clarify conceptual matters before submitting for publication. Thank you for your comments and we appreciative all the feedback we have received from reviewers.

**Reviewer #5**

I want to congratulate the authors for the well-written work on an important topic that might influence the management of ADHD. I have a few comments that I hope will improve the paper. Thank you for your feedback and suggestions.

- 1. I would suggest including a distinct subheading to present the study's conclusion. Perhaps, lines 617-629 could be included in the CONCLUSION section. As requested, we have added a “Conclusion” subheading above the selected section (see line 654).
- 2. Furthermore, references lack complete data, such as DOIs. Providing this information is crucial to facilitating readers' access to the cited documents. It is essential to ensure that all references, including DOIs, are complete to enhance the comprehensiveness and accessibility of the bibliography. Thank you for this suggestion. We have now included the DOIs where available.

**Reviewer #6**

**Abstract**

Though the abstract is good but the following need to be addressed

- One introductory statement (A brief introduction) is necessary to put the study into perspective. Just one sentence or two at most. An additional sentence has been inserted at the start of the Abstract to put the study into additional perspective (see lines 23-25).
- In line 23-just as PA has been written in full, it would be good to first write ADHD in full then abbreviate later. This has been addressed (see lines 23-24).

**Introduction**

- The introduction is clear and straight on point but in line 58-61 the authors have mentioned the challenges that arise as a result of ADHD but they have failed to mention the symptoms making the section unclear. The opening paragraph of the introduction covers areas of function that may be compromised with ADHD, and we have also now inserted additional mention of these symptoms (e.g., inattention, impulsivity, poor decision making, working memory problems) at the suggested point in paragraph two (see lines 59-60).
- The statement of the problem is clearly explained and is nicely linked to the purpose of the study. Thank you for your comments.

**Methodology**

- The methodology is relevant, the inclusion and the exclusion criteria are quite clear.

**Data Processing**

Line 266- Be specific which test statistic was used to analyze which data? To avoid creating potential confusion, we have removed the previous subheadings within section ‘2.5 Data Processing and Statistical Analysis’. In hindsight, we appreciate that the original subheadings within section 2.5 (e.g., Descriptive Statistics; Relationship between PA Level and ADHD Symptomology) may have led to the expectation that specific outcomes and results would be presented at that point rather than within the Results section that follows. Additionally, we have also made reference to each hypothesis throughout section 2.5, allowing the reader to more easily follow the statistical approach (and tests) used to address each (e.g., see lines 286-287, 292-294, 301-305).

**Descriptive statistics**

- 2.5.1 – The authors should come out clear, is these inferential statistics or descriptive? This should be addressed. As mentioned above, we have removed the previous subheadings within section ‘2.5 Data Processing and Statistical Analysis’. In hindsight we appreciate that the original subheadings within section 2.5 (e.g., Descriptive Statistics, Relationship between PA Level and ADHD Symptomology) may have led to the expectation that specific outcomes and results would be presented at that point.
- Line 339- is it conducing or conducting? This has been corrected (see line 335).

**Analysis of results**

- The authors used quantitative analyses and have clearly explained how they used each of the test statistic but what hypothesis was being tested? It is important for a quantitative study to have the hypothesis/ hypotheses stated.
- The findings that the study produces are very critical but have no implications, and they only need one or two sentences to bring out the implication of each finding.
- The manuscript is well written with key subtopics availed. The authors however need to be more interpretive in data presentation. The results are well analyzed and presented but have not been interpreted at all. Making it difficult to make exact meaning. It is always good practice to interpret all findings, this makes it easy to come up with the implications of the findings. The manuscript would be more useful to a broader readership if the authors moved from just providing results to interpreting them and giving the implication as well.
- Generally, the whole section dealing with results needs further explanation and expansion.

Thank you for your feedback and suggestions regarding the analysis of results. We have referred to each hypothesis throughout section 2.5, allowing readers to more easily follow the statistical approaches and tests used to address each of our hypotheses (e.g., see lines 286-287, 292-294, 301-305). Complementing this detail, as well as the reporting of hypotheses at the end of the introduction section, we have also inserted further references to the specific hypotheses being tested within the results section itself (e.g., see lines 334-335, 391-392, 419-420, 426-427). We have also inserted two new subheadings (see lines 418 and 424), with the four subsections 3.2 to 3.5 corresponding to the four hypotheses of the study. Further, we have also inserted reference to the main implication (take-away message) of each main analysis in the results section so that implications are more salient (e.g., lines 355-356, 378-380, 401-403, 422-423, 438-440). Findings are then critically discussed and interpreted throughout the discussion section of the manuscript, further supporting a broader readership to understand the core results and their potential implications.

**DISCUSSION**

The scope of the study is well provided and the presentation is consistent throughout the manuscript. The manuscript is well written and nicely presented quantitatively. This manuscript presents a balanced assessment of key constructs and shows evidence of being well researched, the following areas however need to be addressed;

- The discussion section should start with an objective or hypothesis of the study, this lays a good background for discussion.
- Avoid having recommendations in the discussion section.
- I tend to think that the authors oversimplified the findings thus inhibiting the deeper message that the subject under study could provide yet this is a very important study that can easily influence policy.
- If this study were to be replicated in a different population with similar parameters, would it yield similar/ consistent result? Authors should mention whether the study is generalizable and the gaps identified.
- Discussion should include current findings and relate it to past findings or similar studies elsewhere, this has not been done exhaustively.

Thank you for your comments on the Discussion section, including your positive feedback. To lay further background for the discussion and to serve as a reminder for the reader, we have elaborated on the main objectives/aims of the study at the start of the discussion section. While the main findings are briefly outlined in the opening paragraph, they are discussed in more depth in the sections that follow, with discussion organised by hypothesis (see lines 464, 498, 529 and 551 as examples). Thank you for also recognising the potential impacts of our work, including potential policy implications. We have endeavoured to make our reference to the possible policy and practice implications of our work more explicit (e.g., lines 576 and 600-602). We have also made specific reference to the generalisability of our findings in the ‘limitations and future directions’ section of the manuscript (line 607), with this section as a whole also serving to highlight gaps in our understanding and considerations for future work in the area. Finally, we have further bolstered reference to prior literature and findings throughout the discussion section (e.g., lines 467-469, 477-481, 517-519, 534-539).

**Recommendations**

- This section is good but should be refined to avoid repetition. Thank you, we have streamlined wording in the ‘limitations and future directions’ section to avoid redundancy and/or repetition.

**Conclusion**

The conclusion is well written and is touching on the subject area. Secondly, conclusions are made based on the findings and discussion so avoid mixing this with recommendations, move all recommendations to the section designated for this. Authors should look at the aim of the study and conclude appropriately stating whether the objective(s) for the study was/ were achieved or not. We have moved the mention of specific recommendations further up in the discussion (limitations and future directions) and now focus on broader points in the conclusion – including the central aims of the study (lines 660-667)

**References**

- These are well written Thank you, and we have now included the DOIs where available also.

**Minor Issues**

- The study needs quick edit, there are a few mistakes on typos, tenses and spellings but generally, it is a good manuscript. Apologies for this oversight. We have since identified and corrected some small spelling and grammar issues throughout the manuscript body.
